# Evaluation of Stakeholder Assessment and Engagement Techniques for Incorporation Into Structured Decision‐Making Processes

**DOI:** 10.1002/wer.70187

**Published:** 2025-10-10

**Authors:** Daehyun Ko, John W. Norton, Glen T. Daigger

**Affiliations:** ^1^ Ministry of the Environment, Republic of Korea Sejong‐si Republic of Korea; ^2^ Great Lakes Water Authority Detroit Michigan USA; ^3^ University of Michigan Ann Arbor Michigan USA

**Keywords:** decision‐makers, decision‐making, management, stakeholders, wastewater

## Abstract

Incorporating the priorities and values of relevant stakeholders and decision‐makers is essential for effective wastewater management decision‐making to obtain results that are supported by the relevant parties and, consequently, can be implemented. Here, we report results from evaluations of stakeholder assessment and engagement techniques in real‐world contexts to further evaluate a decision‐making process previously developed as part of a broader research program for the Great Lakes Water Authority (GLWA), the regional water and wastewater utility serving 4 million people in Southeast Michigan. Two important decisions to be made by the GLWA, namely, (1) improvements to biosolids management and (2) upgrades to their bar racks and grit chambers, provided the basis for this assessment. We found that structured interviews of potential stakeholders and decision‐makers produce useful information to identify relevant parties and determine decision goals, objectives, and criteria. Identification of primary responses and keywords for each of the survey questions, followed by an affinity analysis to identify key themes and outcomes of the interviews, provided useful results. Multicriteria decision analysis (MCDA) integrates the priorities and values of stakeholders and decision‐makers and enhances interactions between stakeholders and decision‐makers, increasing knowledge transfer, encouraging the discovery of new perspectives, and helping stakeholders to consider new alternatives or combinations of existing options. Eliminating inferior alternatives during the screening and detailed evaluation phases increased clarity and efficiency. Tools such as data envelope analysis (DEA), analytic hierarchy process (AHP), simple additive weighting (SAW), and technique for order of preference by similarity to ideal solution (TOPSIS) were found to supplement technical analyses with the priorities and values of various stakeholders and decision‐makers.

## Summary

- The priorities and values of relevant decision‐makers and stakeholders must be incorporated into wastewater management decision‐making processes to achieve supported and implementable solutions.
- A variety of tools are available to assess priorities and values and incorporate them into the decision process including multicriteria decision analysis (MCDA), data envelopment analysis (DEA), analytic hierarchy process (AHP), process modeling, economic assessments, life cycle assessment (LCA), and social life cycle assessment (SLCA).
- Use of these tools, where appropriate, can effectively assess the priorities and values of the diverse range of decision‐makers and stakeholders for incorporation into the decision process.
- MCDA provides a useful framework for integrating decision‐maker and stakeholder priorities, along with supporting technical evaluations, into an effective decision‐making process.

## Graphical abstract

A variety of tools were evaluated and found to be effective in assessing the priorities and values of decision‐makers and stakeholders and incorporating them into the wastewater management decision process. Integration of priorities and values with supporting technical evaluations through MCDA results in supported and implementable solutions.

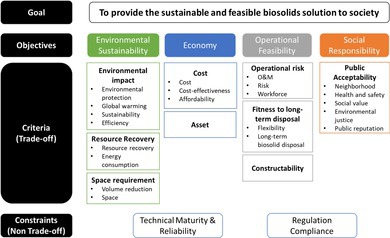

## 1 Introduction

While decision‐making for wastewater management systems may be viewed by some as a simple technical exercise, it is a complex process requiring various levels of information and participation of multiple decision makers and stakeholders to inform and influence organizational decision‐making. Prior to the 1990s, the process involved a limited number of decision makers and experts, with economic and technical aspects as the primary focus (Logan et al. 1962; Hipel 1992; Matthews et al. 2012). However, in recent years, environmental and social aspects have been increasingly emphasized, and participation has been broadened to encompass a diverse range of stakeholders, including utilities, design engineers, federal, state, and local regulators, local officials, residents, nongovernmental organizations (NGOs), and other stakeholders (Erb‐Institute 2017, 2021; USEPA 2012, 2015). The application of structured decision analysis in the water sector is necessary to make the increasingly complex decision‐making process more efficient and thorough, leading to decisions that are supported by the relevant parties and, consequently, can be implemented. Methods such as stakeholder analysis to identify relevant participants in the decision process and multicriteria decision analysis (MCDA) to support decision‐making from various perspectives can be incorporated into the decision‐making process (Chrispim et al. 2020; Ko et al. 2024; Lienert et al. 2011; Lizot et al. 2020).

**FIGURE 1:**
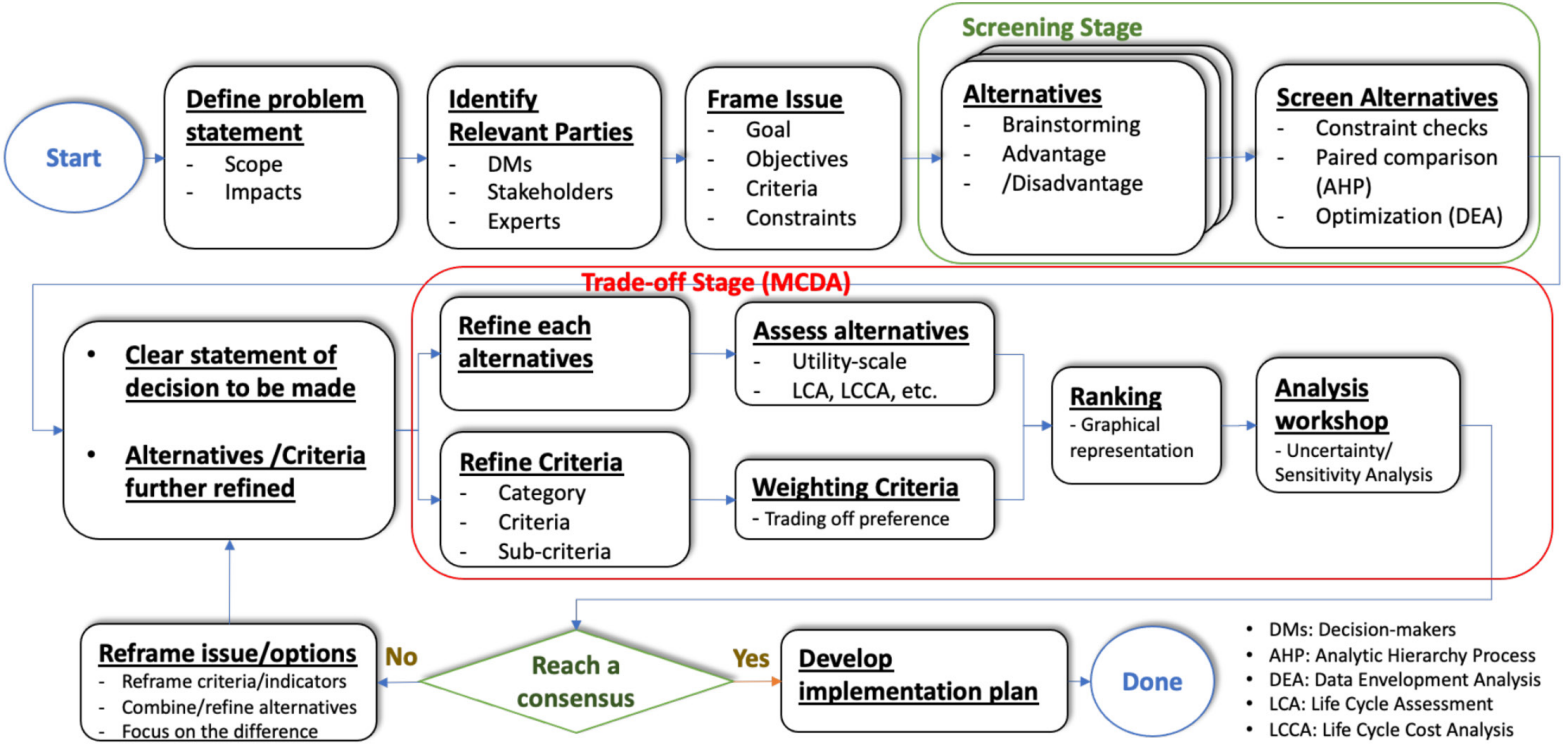
Wastewater management decision process (Ko et al. 2024).

The Great Lakes Water Authority (GLWA) provides water and wastewater service for much of Southeast Michigan, serving roughly four million water and three million wastewater customers. As one component of a broad assessment of decision‐making approaches for GLWA (Ko et al. 2025), we developed a wastewater decision‐making process, illustrated in Figure 1, that is based on sustainability principles and that integrates technical evaluations and stakeholder priorities and values (Ko et al. 2024). The first step of the previously developed process is to define the decision problem and identify the relevant decision makers and stakeholders. Decision‐makers are defined as “the combination of individuals who have the organizational authority to direct the actions necessary for the decision to be implemented”. Stakeholders are defined as “individuals or representatives of formal and informal groups affected by the outcome of the subject decision and who can influence its subsequent implementation.” A structured approach, such as stakeholder analysis, can be used to identify the decision makers and stakeholders. The decision makers and stakeholders then explore goals, objectives, criteria, and constraints as they progress through the screening stage, where the broad range of options is evaluated, and the trade‐off stage, where the most viable options are evaluated in more detail. MCDA is commonly used to integrate the diverse values and priorities of the decision makers and stakeholders with the technical evaluations that inform the decision process. The process is iterative and involves reframing the issue and investigating new options if consensus is not reached. The process is complete when a path forward is determined that (1) the relevant decision‐makers and stakeholders can support and (2) an implementation plan has been developed. We subsequently demonstrated how wastewater process modeling can be integrated with life cycle analysis to support technical‐economic evaluations and assess the environmental impacts of the options under consideration (Ko et al. 2025).

Effective stakeholder engagement is an essential component of the decision‐making process, illustrated in Figure 1, to identify their priorities and values and integrate them into the decision process. We had previously identified a variety of potentially useful stakeholder engagement techniques (Ko et al. 2024), but our previous research had not provided an in‐depth understanding of their practical utility and effectiveness in actual practice. Consequently, research was initiated using two different wastewater management decisions facing GLWA, namely wastewater biosolids management and improvement to their bar racks and grit chambers.

**FIGURE 2:**
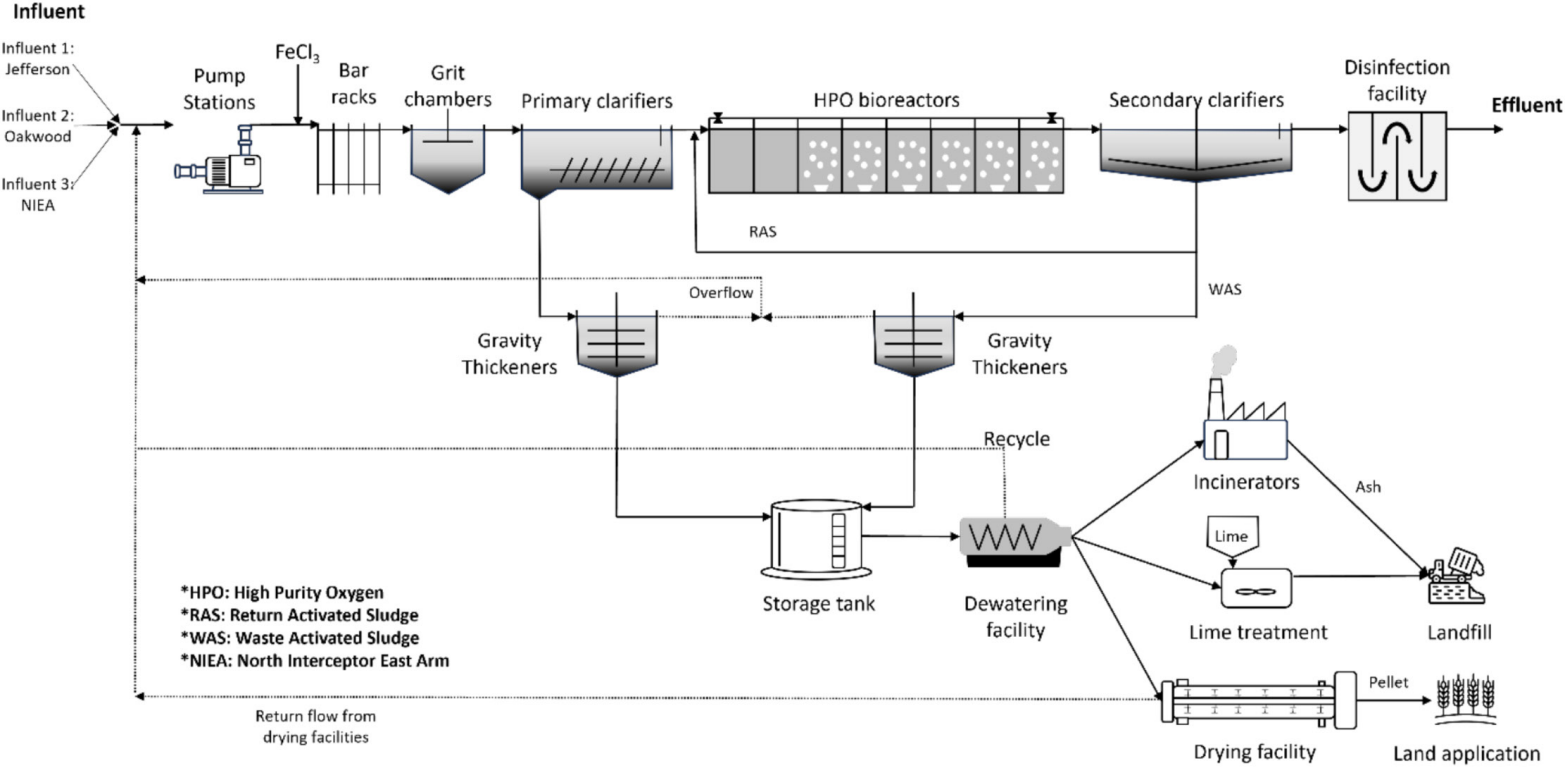
GLWA WRRF.

The GLWA water resource recovery facility (WRRF) (Figure 2) is a secondary treatment facility with a seasonal monthly average effluent total phosphorus limit of 0.7 mg‐P/L (0.6 mg‐P/L during April through September). The liquid process train consists of influent pumping, preliminary treatment with ferric chloride addition for phosphorus removal, primary treatment, high‐purity oxygen activated sludge incorporating biological phosphorus removal, followed by disinfection prior to discharge to the Detroit River. The GLWA WRRF services a combined service area with peak sustained treatment capacity of 6,400,000 m^3^/day (1700 mgd) and secondary treatment capacity of 3,600,000 m^3^/day (940 mgd). The biosolids process train consists of separate gravity thickening of primary and waste activated sludge (WAS) and storage prior to processing through either a dewatering and drying facility, incineration, or lime stabilization. An average of approximately 400 tons of solids is processed each day. Approximately three‐quarters of the solids are processed through the relatively new (~10 years) dewatering and drying facility, the remainder through the more than 60‐year‐old incineration facility, and lime stabilization as a seldom‐used back‐up. The extended life of the incineration facility, coupled with upcoming more stringent air quality control requirements, necessitates the replacement of the incineration facility. Significant modifications to their wastewater biosolids management system are needed to (1) address aging infrastructure (incinerators), (2) minimize the amount of biosolids landfilled, and (3) reduce greenhouse gas (GHG) emissions compared to the existing baseline.

The GLWA WRRF preliminary treatment system consists of mechanically cleaned bar racks with 0.75‐in. openings, followed by aerated grit chambers with settled grit removed through a clamshell bucket and then transported to landfill (Hazen and WadeTrim 2021). These facilities have reached their useful life, and significant grit loads during rain events from the combined sewers result in grit loads that the system is not able to properly handle. Improvements to the bar racks and grit chambers were needed to (1) improve screening and grit removal efficiency, (2) increase long‐term system reliability, and (3) simplify operation and maintenance (O&M).

The management and operations of both the biosolids treatment system and the grit removal system place a heavy burden on staff, especially during rain events, due to the system design and historical maintenance of the infrastructure. These challenges informed the GLWA decision‐making process with locally specific drivers and reliability outcomes. Other utilities will inherently develop different solutions, based on their own unique circumstances.

Here, we report results from our evaluation of the effectiveness of a variety of stakeholder assessment and engagement techniques when applied to these two GLWA decisions in the context of the broader decision‐making process described above. The overall objective is to gain experience and insights into the use of a variety of stakeholder assessment and engagement techniques within a real‐world context. At the time these evaluations were conducted, GLWA was formulating its approach to biosolids management, including preparing to issue a request for proposals (RFP) to select a consultant to conduct a biosolids feasibility study. We interviewed internal stakeholders, performed an initial stakeholder assessment, and identified parties relevant to the decision process, along with a hierarchy of decision goals, objectives, and criteria, which informed the development of the biosolids master plan RFP. Evaluation of bar rack and grit chamber options was ongoing. We also found that techniques such as data envelope analysis (DEA) can supplement MCDA to incorporate stakeholder and decision‐maker priorities and values and foster social learning and collaboration to identify solutions that can be broadly supported and implemented. The interviews were conducted in writing or using virtual meeting platforms, as this assessment was largely conducted during the COVID‐19 shutdown.

## 2 Methods

### 2.1 Biosolids Decision Assessment

The research team collaborated with GLWA staff to help frame the key biosolids management decisions to be made by GLWA and to assess the use of stakeholder tools to assist with this framing. An initial stakeholder analysis for biosolid management decisions was conducted in consultation with GLWA staff. The procedure from the Erb‐Institute (2021) was used, which consists of three steps:

1. Define the scope.
  - Define organizational boundaries
  - Define operational boundaries
  - Define geographical boundaries
2. Identify the issues and impacts.
  - Economic
  - Environment
  - Social
3. Generate a list of stakeholders.
  - People
  - Place
  - Process
  - Products/services

The identified stakeholders were then mapped onto a two‐by‐two matrix consisting of impact and importance, with each ranging from low to high (Figure S1). The location of an individual stakeholder on the matrix is determined by their importance to the outcome of the decision process and the impact of the decision on them (Grimble and Wellard 1997; Lienert et al. 2013; Reed et al. 2009).

In consultation with GLWA members of the research team, and reflecting the mapping results above, it was decided to conduct the first round of interviews with internal stakeholders to more clearly define stakeholder groups for subsequent interviews. Only internal (GLWA) stakeholders were interviewed because of the confidential nature of the early stage of development of the Biosolids Master Plan RFP. Nine interviewees were selected from various teams, including operation and maintenance (O&M), engineering, finance, and construction, to reflect the diverse perspectives and values of the internal stakeholders within GLWA. The interview protocol was reviewed and approved by the University of Michigan Institutional Review Board (IRB). The selected interviewees were then briefed before the interview on the overall decision‐making process illustrated in Figure 1 and the biosolid management decision. The interview questions were mostly open‐ended, as shown in the survey form included in the Supporting Information. The questions covered the following topics: framing issues, the importance of the decision, identifying relevant parties, criteria, alternatives, and further information. The interviews lasted about 1–2 h each and were conducted virtually via online meeting and were then transcribed. Primary responses and keywords for each of the survey questions were identified, followed by an affinity analysis to identify key themes and outcomes of the interviews. Similar items from the extracted keywords were clustered into groups, which were organized into broader categories. Affinity diagrams were used to present the grouped keywords and their respective categories (after Brassard and Ritter 2018). Interview responses and the extracted keywords are presented in the Supporting Information. A summary of responses and keywords is provided, along with the affinity analysis for select questions. These results were used to formulate a draft hierarchy of goals, objectives, and criteria and a revised assessment of parties relevant to the biosolids decision.

### 2.2 Bar Rack and Grit Chamber Decision

The bar racks and grit chambers were to be upgraded to (1) improve screening and grit removal efficiency, (2) increase long‐term system reliability, and (3) simplify O&M. The decision criteria being used in this evaluation by GLWA and their consultant were: (1) the amount of SS removed, (2) construction cost, (3) operation cost, and (4) social score. The social score was calculated through internal staff interviews using the paired comparison technique (PCT) for categories (1) performance/reliability, (2) regulation, (3) O&M, (4) health and safety, (5) public benefit, and (6) sustainability. Some alternatives were excluded from consideration due to constraints in O&M or hydraulic limitations (Hazen and WadeTrim 2021). The evaluation process was reaching its final stage at the time the analysis reported here was conducted. The objective of the study reported here was to retrospectively analyze the previously conducted evaluation process from a new vantage point to identify opportunities for improved stakeholder engagement and the potential effectiveness of various stakeholder engagement tools.

The GLWA members of the research team identified four internal GLWA staff who were involved in the operation and engineering of the bar racks and grit chambers or who were engaged in the decision process to participate in the interviews. Given the objective of the retrospective analysis, the following questions were asked about their experience and perception of the decision.

1. Background question: the reason for participation, role, and responsibility in the decision, and key interests
2. Framing issue: decision goals, objectives, and constraints
3. Identifying criteria: decision criteria and satisfaction
4. Weighting criteria: AHP analysis
5. The final decision: identification and assessment of the final decision
6. Other relevant parties: identification of other decision‐makers and stakeholders

The specific interview questions are presented in the Supporting Information. The interviews were conducted virtually via online meetings, and each extended for a duration of 60–90 min. The interview protocol had prior review and approval from the University of Michigan Institutional Review Board.

Various tools were found to be useful for evaluation of the decision analysis of the bar rack and grit chamber. DEA was used as a screening tool, and the analytic hierarchy process (AHP) was applied for criteria weighting. For MCDA ranking methodologies, simple additive weighting (SAW) and Technique for Order of Preference by Similarity to Ideal Solution (TOPSIS) were utilized.

DEA is a methodology for measuring the efficiency of data with multiple inputs and outputs (Charnes et al. 1978). Within the methodology, alternatives used to compare performance are referred to as Decision‐Making Units (DMUs). Based on the supplied inputs and the produced outputs, DEA differentiates efficient and inefficient alternatives through relative comparison among DMUs and is, thus, a “weight‐free” method of screening and prioritizing alternative DMUs. The Banker, Charnes, and Cooper (BCC) input‐oriented DEA model in the DEA Solver LV 8.0 software (Open Source). The analysis was conducted using variable returns to scale for 17 DMUs. Construction and operation costs were applied as input variables, while the output variables were the amount of SS (suspended solids) removed and the social score (Hazen and WadeTrim 2021).

AHP is a decision‐making method developed by Saaty and is commonly used for determining the criteria weighting (Saaty 1987). Through paired comparisons between criteria, the relative importance of each criterion can be identified. In addition, a consistency check allows the validity of the response outcomes to be assessed. In this study, three respondents had consistency ratios below 0.1, indicating valid results. However, one respondent had a consistency ratio exceeding 0.1, so an alternative tool—the reverse swing method—was used to determine the weights (Lienert et al. 2011; Schuwirth et al. 2012).

For MCDA methodologies, SAW and TOPSIS, which are commonly used in WRRFs, were applied (Kalbar et al. 2015). SAW is a method used to determine rankings by comparing the total sum of weighted scores for each alternative. TOPSIS is used to find the best alternative by identifying the one with the shortest geometric distance from the ideal solution and the farthest distance from the anti‐ideal solution. Thus, it allows alternatives to be ranked based on their proximity to the ideal and anti‐ideal solutions.

## 3 Results

### 3.1 Biosolids Decision Assessment Results

An initial structuring of the Biosolids Master Plan was first conducted to identify the stakeholders to be interviewed. The results of the interviews are then presented. These results provided the basis to formulate an initial hierarchy of the goals, objectives, and criteria for the decision process and to refine the relevant parties to be involved in the process.

#### 3.1.1 Initial Structuring of Biosolids Master Plan

Table 1 presents the results of the initial stakeholder analysis. The organizational boundary was defined as GLWA, and the operational boundaries were defined as biosolids management at the GLWA WRRF. Related biosolids management suppliers and supply‐chain partners were identified, such as the owner/operator of the biosolids drying facility (BDF), which processes approximately three‐quarters of the biosolids produced at the WRRF, the landfill occasionally used for biosolids disposal, and the parties responsible for land application of the pellets produced at the BDF. The geographical boundaries were set as GLWA WRRF, local communities, and the farmland where the biosolid pellets are applied.

**TABLE 1:** Initial stakeholder analysis.

Step	Analysis process	
1. Define the scope	Organizational boundaries: GLWA	
	Operational boundaries	Biosolid management: thickening, dewatering, and incineration
		Related suppliers and other supply‐chain partner: drying (BDF), landfill, and land application
	Geographic boundaries: GLWA WRRF, local communities, and farmland where the dry biosolid pellets are applied	
2. Identify the issues and impacts	Economic: cost, revenue from the recovered resources (fertilizer, energy), expense for local suppliers, economic development, and so on	
	Environmental: solid waste, resource recovery, energy consumption, GHG (Greenhouse Gas) emission, toxic pollutant, and so on	
	Social: labor, noise, traffic, odor, employee safety and health, community engagement, and so on	
3. Generate a list of stakeholders	People: employees, contractors, farmers, nongovernmental organizations (NGOs), regulators	
	Place: local communities	
	Process: operation staff, supply chain (electricity, energy, traffic, landfill, and so on)	
	Products/services: customers	

The decision issues and impacts initially consisted of economic issues, including cost, revenue, and local economic development, environmental issues, including solid waste, resource recovery, energy consumption, and pollutant release, and social issues, including labor, noise, traffic, odor, safety and health, and community perception.

**FIGURE 3:**
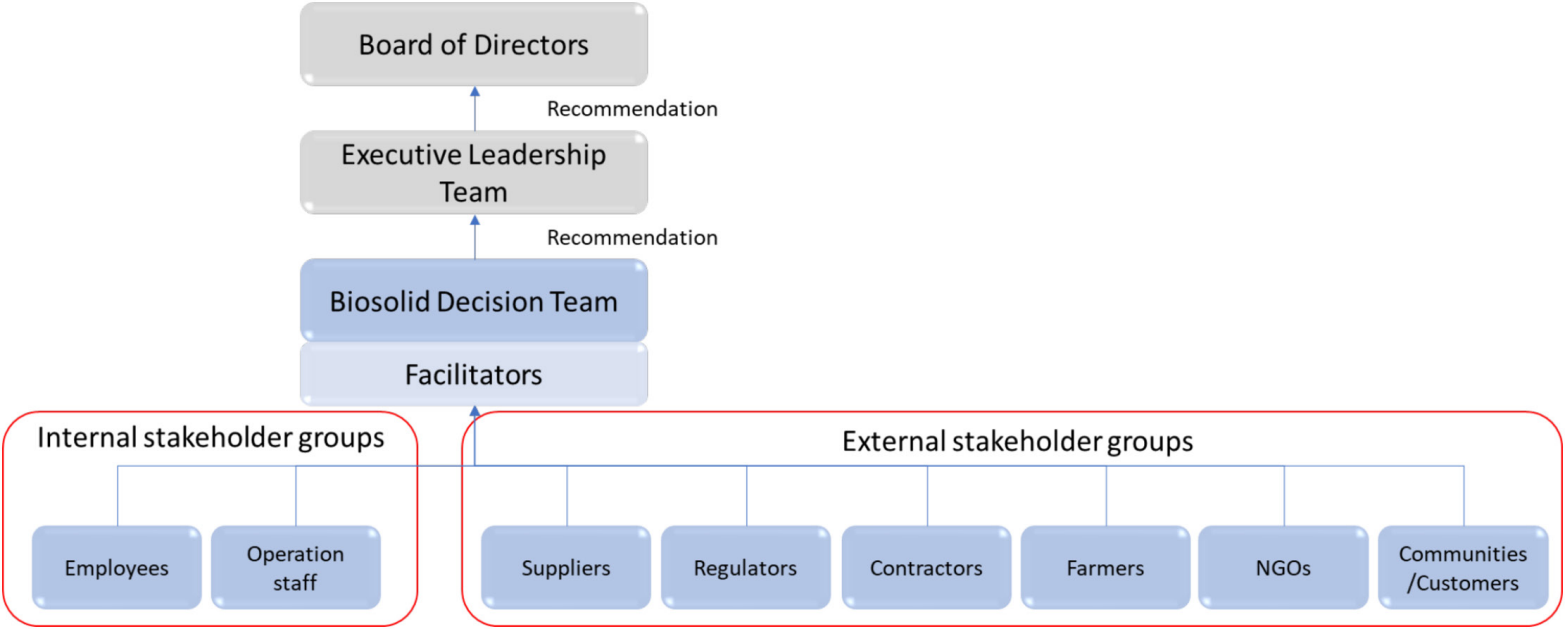
Preliminary biosolids decision‐making structure.

Figure 3 presents the decision‐making structure initially identified. Decision‐makers and stakeholders are defined above. Subject matter experts (SMEs) apply their varied professional expertise to provide greater understanding of the decision for decision‐makers and stakeholders. Ultimately, a recommendation will be developed and presented to the GLWA Executive Leadership Team (ELT) and subsequently to the GLWA Board of Directors (BoD).

#### 3.1.2 Biosolids Interview Results

**FIGURE 4:**
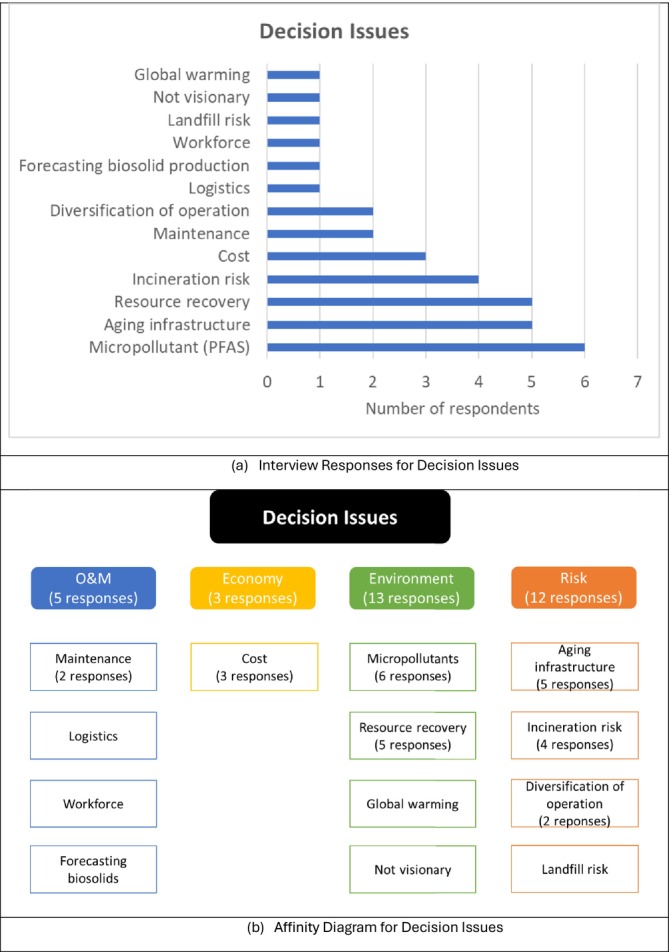
Interview results and affinity diagram for decision issues for GLWA WRRF biosolid management.

With the overall structure established, interviews with the identified internal stakeholders were conducted. Figure 4 summarizes responses concerning current and emerging biosolids management issues relevant to the biosolids management options to be considered by GLWA. The number of respondents addressing each issue is provided in the figure, and the results are organized into affinity groups. There were concerns about micropollutants, as well as significant recognition of the aging infrastructure in the current sludge stream. At the beginning of this research, the state of Michigan had banned the land application of biosolids containing more than 150 μg/kg perfluorooctanoic sulfonate (PFOS), but this was later revised downward to 120 μg/kg PFOS (EGLE 2021). Issues regarding higher O&M costs and heightened regulations for air pollutants as the incinerators approach the end of their useful life were further identified. There were also mentions of a future plan to transform from an energy‐intensive facility into a more sustainable facility through resource recovery.

**FIGURE 5:**
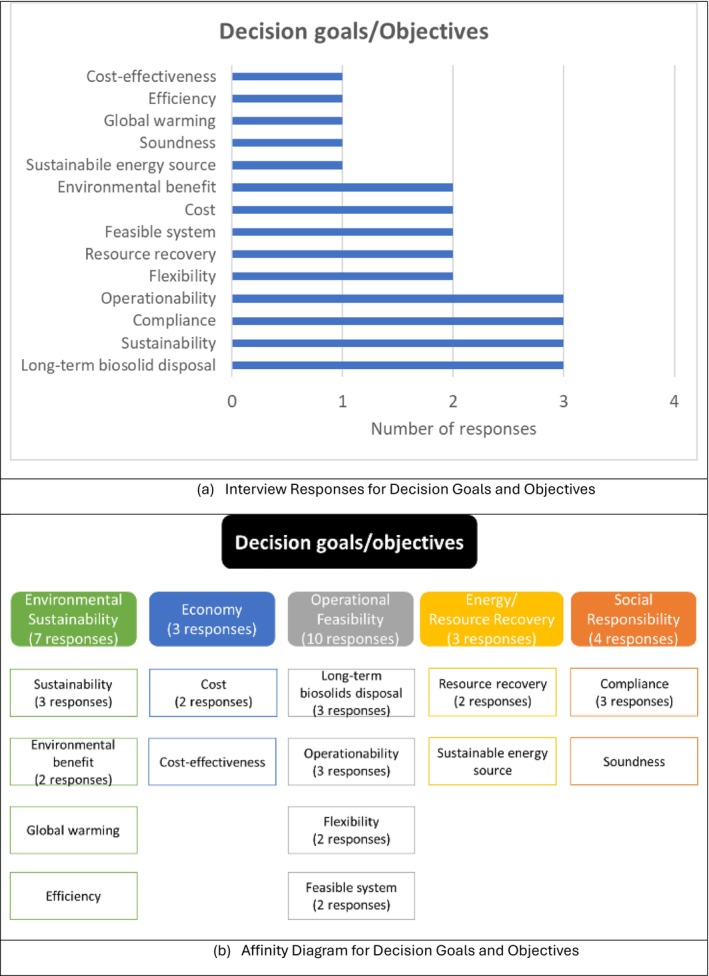
Interview results and affinity diagram for decision goals and objectives for GLWA WRRF biosolid management.

Interviewees were asked two questions to address decision issues and identify the goals of biosolids management decisions: (1) What is the overarching goal of the decision? 2) What do you want to achieve in the decision? To determine the decision objectives, interviewees were asked the following question: “What are the specific objectives of the decision needed to achieve the goal?” The combined interview results for decision goals and objectives are presented in Figure 5. The highest priority was given to long‐term biosolid disposal, sustainability, compliance, and operability, and the second highest priority was flexibility, resource recovery, feasible systems, cost, and environmental benefit. The dominance of operation‐related responses is presumably due to most of the interviewees being internal stakeholders. Internal stakeholders expressed a preference for operability and flexibility. Moreover, they sought solutions to address biosolid disposal from a long‐term perspective and to consider future regulations adequately. Ten responses mentioned operational feasibility, indicating significant interest. The second most frequent responses were in the environmental sustainability category.

**FIGURE 6:**
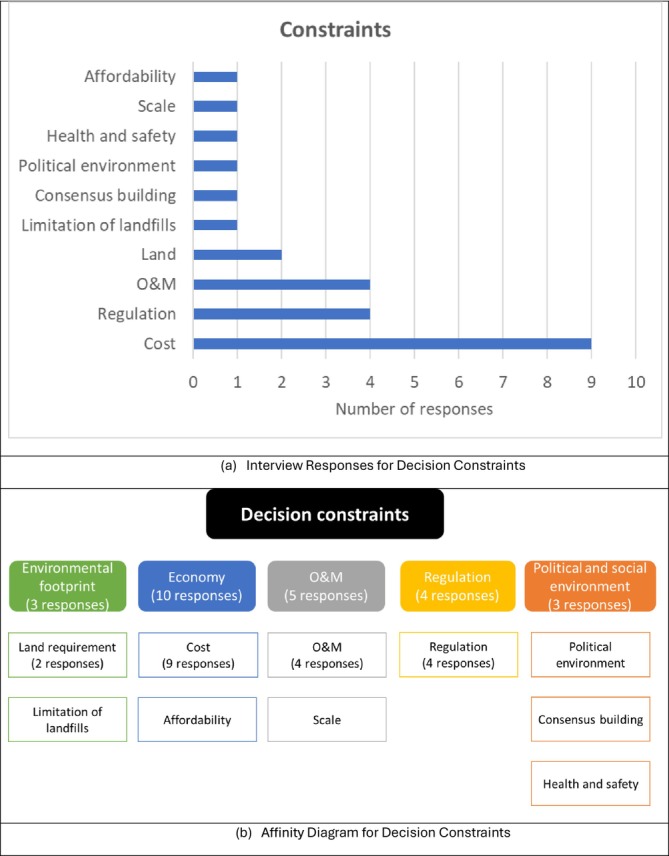
Interview results and affinity diagram for decision constraints for GLWA WRRF biosolid management.

Responses concerning constraints are presented in Figure 6. Nine interviewees identified cost as a constraint, and four cited regulations and O&M as constraints. Additionally, constraints such as land requirements, limitations of landfills, consensus building, political environment, health and safety considerations, scale, and affordability were identified. The results were organized into five categories. The most frequently mentioned constraints were in the economic category of cost and affordability. In the O&M category, stakeholders wanted ease of operation, a lack of nuisance, and robustness for the biosolid management systems. Additionally, interviewees anticipated that compliance with future strengthened regulations would become a constraint.

**FIGURE 7:**
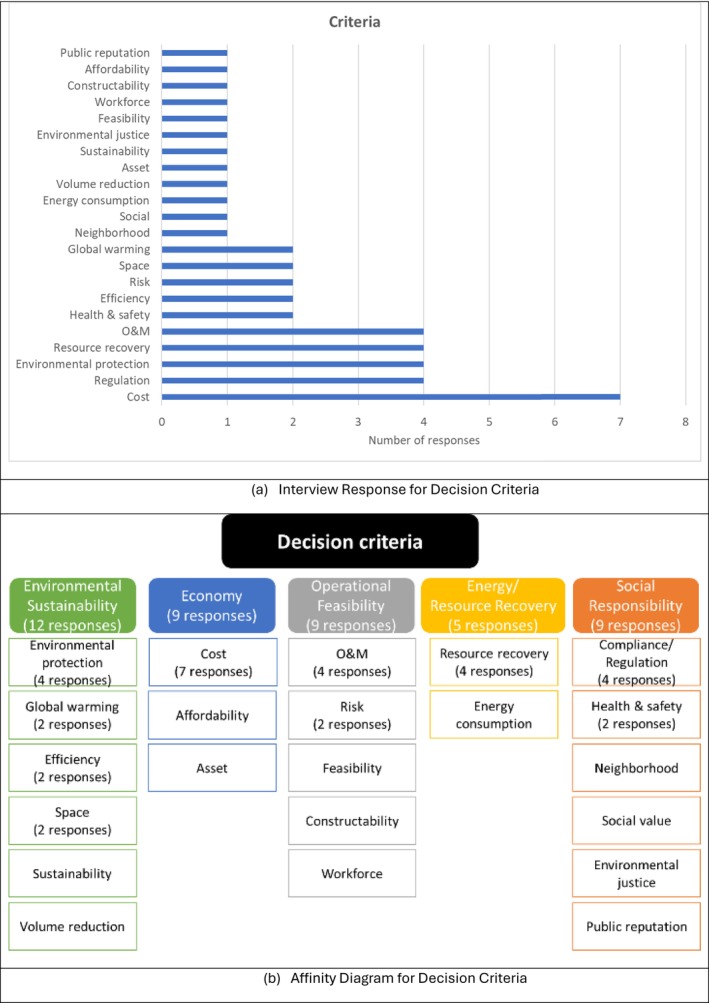
Interview results and affinity diagram for decision criteria for GLWA WRRF biosolid management.

The criteria need to be relevant to the decision goals and objectives, and should highlight differences between the alternatives being considered. Figure 7 presents the identified criteria. Seven out of 9 respondents identified cost as a criterion, while 4 out of 9 identified regulatory compliance, environmental protection, resource recovery, and O&M. Other criteria included health and safety, efficiency, risk, space, and global warming. The identified criteria were grouped based on the triple bottom line of sustainability: environmental, economic, and social aspects. Two additional criteria categories were identified, including operational feasibility and resource recovery. While the environmental sustainability category was mentioned most frequently, the categories are not mutually exclusive and can overlap. For example, sludge volume reduction contributes to environmental sustainability but can also be categorized under the economy due to its role in reducing sludge disposal costs. A notable outcome is that a significant number of responses were related to the social responsibility category.

**FIGURE 8:**
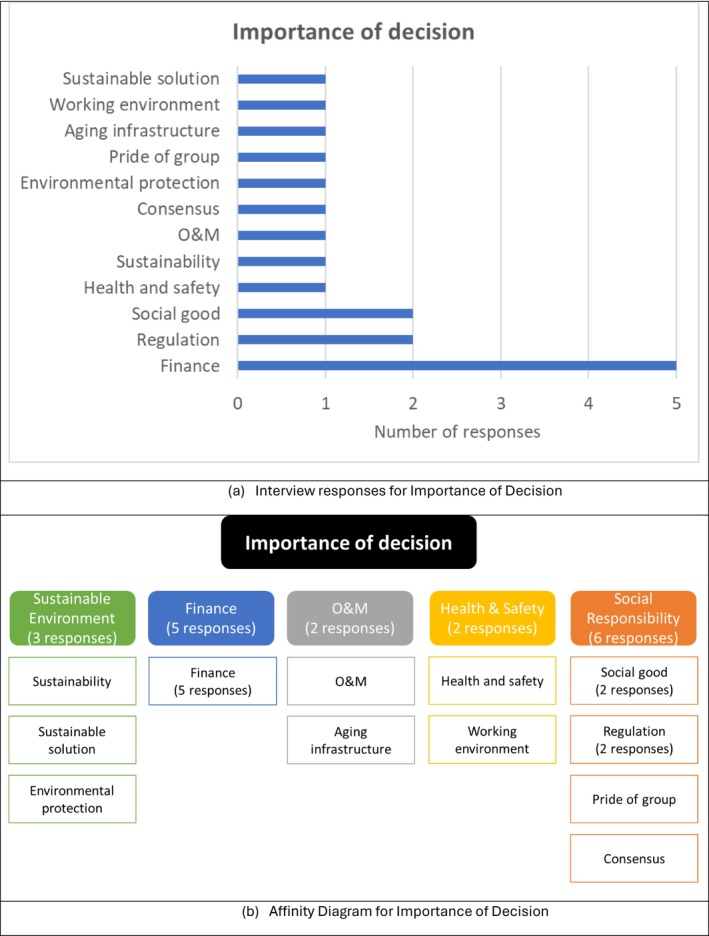
Interview results and affinity diagram for importance of decision for GLWA WRRF biosolid management.

The following questions addressed the importance and impact of the decision: (1) Why is this decision important to you or your group? (2) What impacts could you anticipate? (3) How much could the decision impact you? The responses are summarized in Figure 8. The most common response was that biosolid decisions are financially important due to the high costs associated with biosolid management, followed by the significance of regulatory compliance and the importance of social good, sustainability, health and safety, and O&M. Affinity analysis indicated the importance of biosolid management from the perspective of social responsibility. Biosolid management was shown to be crucial for regulatory compliance and social good due to the significant impact on neighboring communities. These results suggest that future interviews with external stakeholders are recommended due to the importance of external input to the decision process.

**FIGURE 9:**
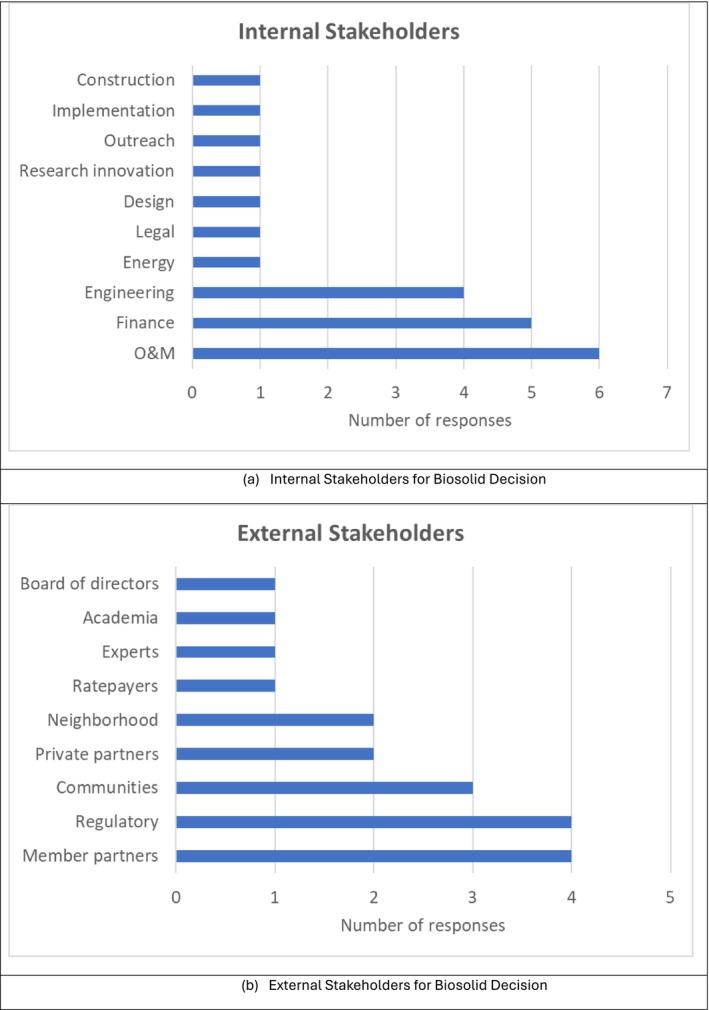
Interview results for internal and external stakeholders for GLWA WRRF biosolid management.

Figure 9 summarizes the internal and external stakeholders identified. Interviewees were asked the following question: “Who are the stakeholders in the biosolid management decision?” Most interviewees identified O&M, finance, and engineering groups as internal stakeholders, but a variety of other groups were identified. External stakeholders identified included member partners (the GLWA term for its customers), regulatory bodies, and communities, along with the surrounding neighbors of the WRRF, and private partners such as New England Fertilizer Company (NEFCO, now owned by Synagro Inc.), which operates the BDF, ratepayers, experts, academia, and the GLWA BoD.

One opinion of note urged that O&M staff be involved in the early stages of decision‐making in order to have more holistic and applicable knowledge.

A lot of times, people who operate the system are forgotten until you have a design and it's done … so, bringing the people who operate the system in on the decision from early on really helps because they will tell you the lessons learned, what works, what doesn't, what to be aware of

#### 3.1.3 Hierarchy of Decision Goals, Objectives and Criteria

**FIGURE 10:**
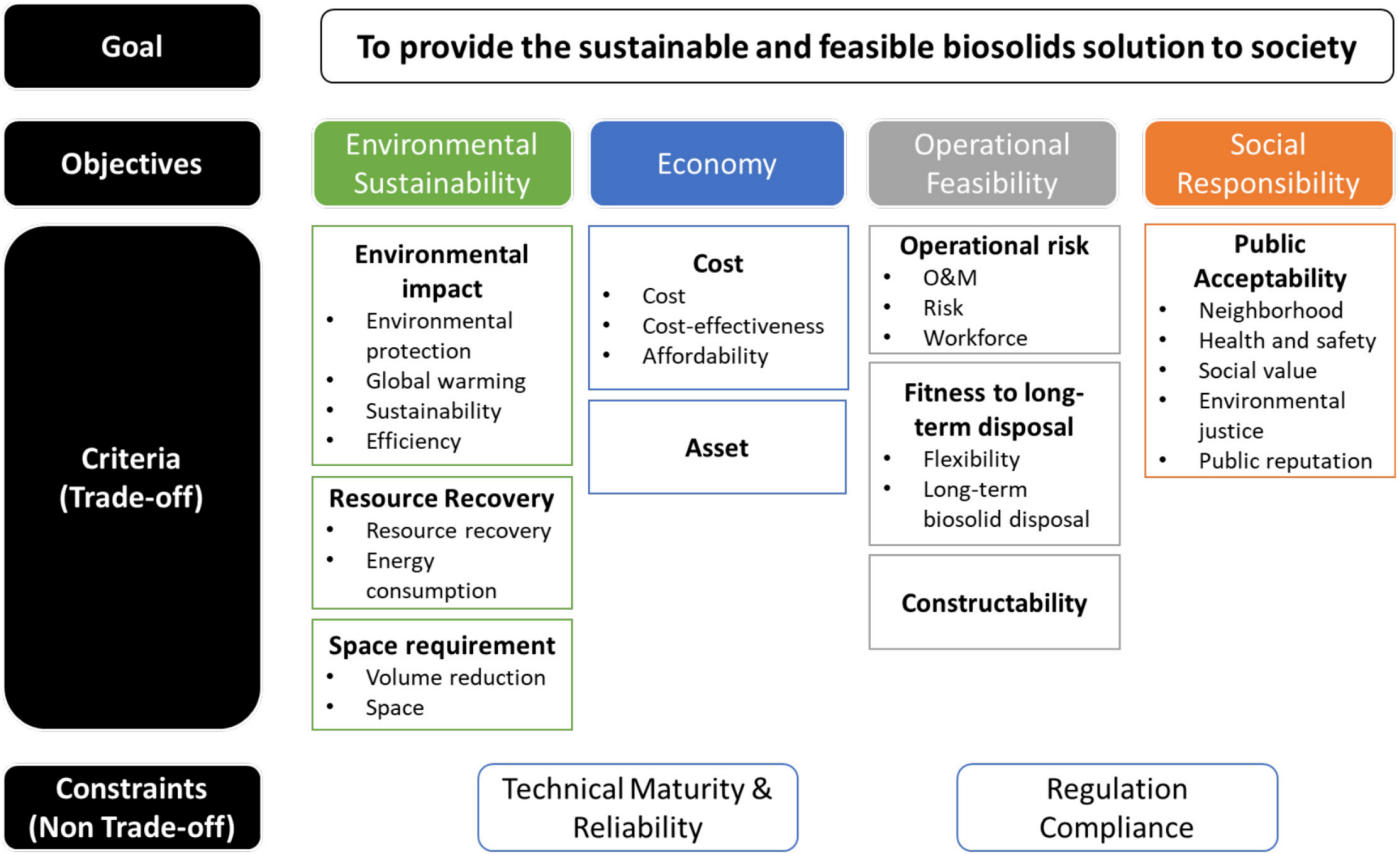
Hierarchy of decision goal, objectives, and criteria.

A hierarchy of goals, objectives, and criteria was created based on the interviewees' responses regarding decision goals, objectives, criteria, and constraints, as shown in Figure 10. A decision goal, “To provide a sustainable and feasible biosolids solution to society,” was formulated based on the frequency of key terms, including sustainability, social responsibility, and a feasible system. By integrating energy/resource recovery into environmental sustainability as a sub‐category, the decision objectives were reduced to four main categories, compared to those shown in Figure 5. Keywords from Figure 3 were consolidated into criteria, while feasible system and compliance were moved to constraints.

While decision criteria and constraints were sometimes used interchangeably by the interviewees, decision criteria refer to items that can be traded off during the decision‐making process, while constraints cannot. The relevant objectives (Figure 5) and constraints (Figure 4) were integrated into criteria and sub‐criteria presented in Figure 10.

#### 3.1.4 Relevant Parties

**FIGURE 11:**
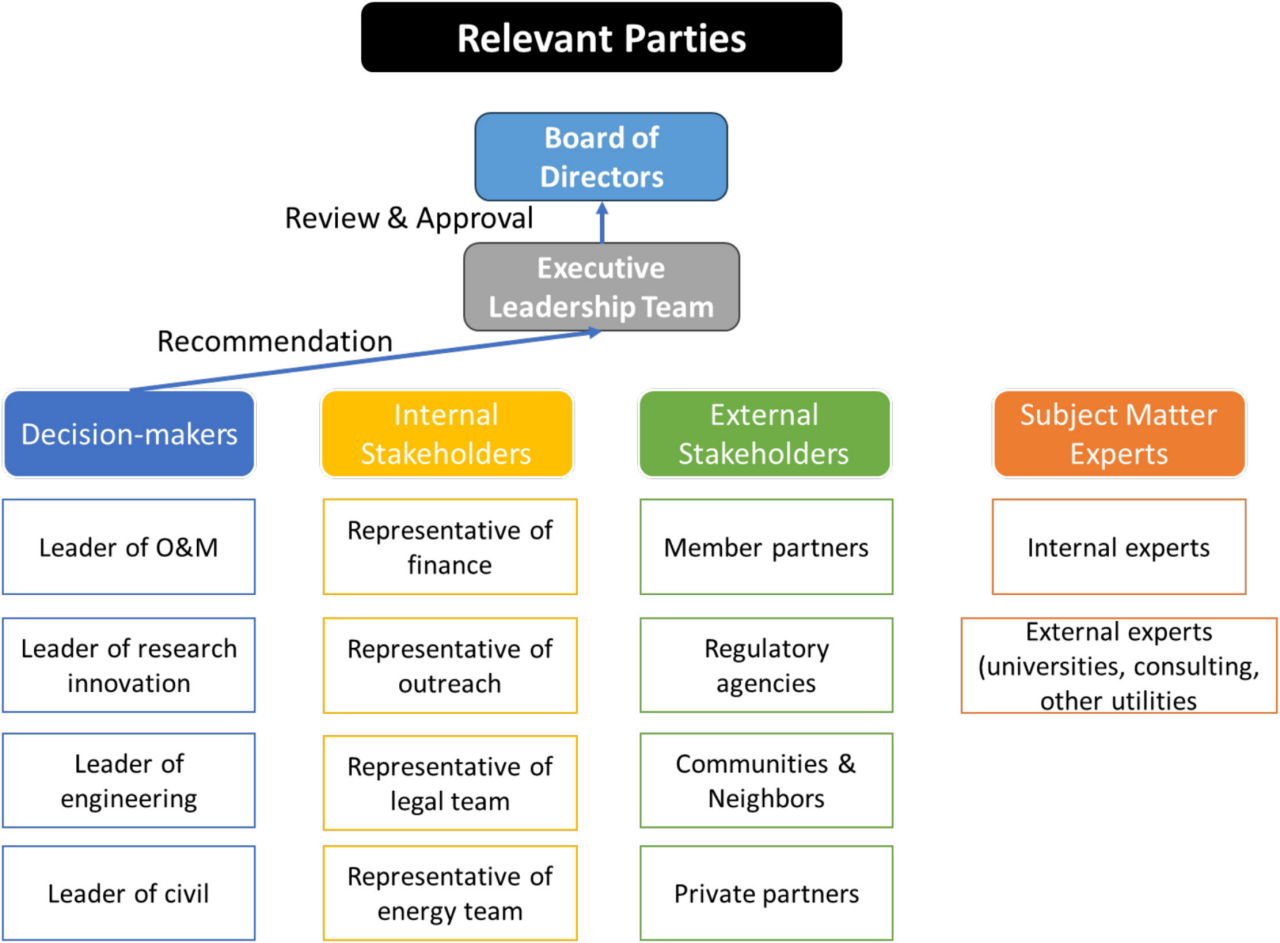
Relevant parties identified from interview results.

Relevant parties were identified based on the interview results, and the preliminary decision‐making structure (presented in Figure 3) was updated, as shown in Figure 11. The decision‐makers include the leaders of the O&M, research, engineering, and planning groups because these groups are directly involved in biosolid management.

Other internal GLWA stakeholders included representatives from the finance, legal, energy, and outreach groups. Internal stakeholder involvement is projected to provide insights into the financial and legal issues related to biosolid decisions. Additionally, since biosolid management significantly impacts the energy balance within the GLWA WRRF, the energy group will provide opinions as an internal stakeholder. In general, the biosolids processing and treatment system consumes a significant amount of energy, while the introduction of technologies such as anaerobic digestion could offset consumption by producing biofuels. The outreach group is expected to facilitate communication and provide information about communities and surrounding neighbors during the decision‐making process.

External stakeholders identified were member partners, regulatory agencies, communities/neighbors, and private partners. GLWA's member partners include counties and cities in the southeastern Michigan area, such as the city of Detroit and Wayne County. The Michigan Department of Environment, Great Lakes, and Energy (EGLE) is the state regulatory agency for this decision. The communities surrounding the GLWA WRRF are also essential external stakeholders. These neighboring communities are easily impacted by the biosolids management decision in various ways, such as noise, pollution, and increased traffic. Additionally, there are NGOs within these communities, including environmental groups, whose opinions should be considered. Private partners may include groups related to sludge transport, treatment, and disposal, such as NEFCO, which operates the BDF.

SMEs can be divided into internal and external experts. Internal experts support decision‐making by applying their specified knowledge as practitioners within GLWA. They offer expertise, particularly in technical and practical issues related to operations. In one interview, it was suggested that professionals from universities, consulting companies, and other national WWTPs should participate as external stakeholders. These professionals would play a key role in the generation and assessment of biosolid alternatives.

### 3.2 Bar Rack and Grit Chamber Decision Assessment Results

The results of the bar rack and grit chamber interviews are presented, along with a sensitivity analysis assessing whether changes in preferences (weights in the MCDA analysis) would significantly impact overall preferences by the individuals interviewed.

#### 3.2.1 Bar Rack and Grit Chamber Interview Results

**FIGURE 12:**
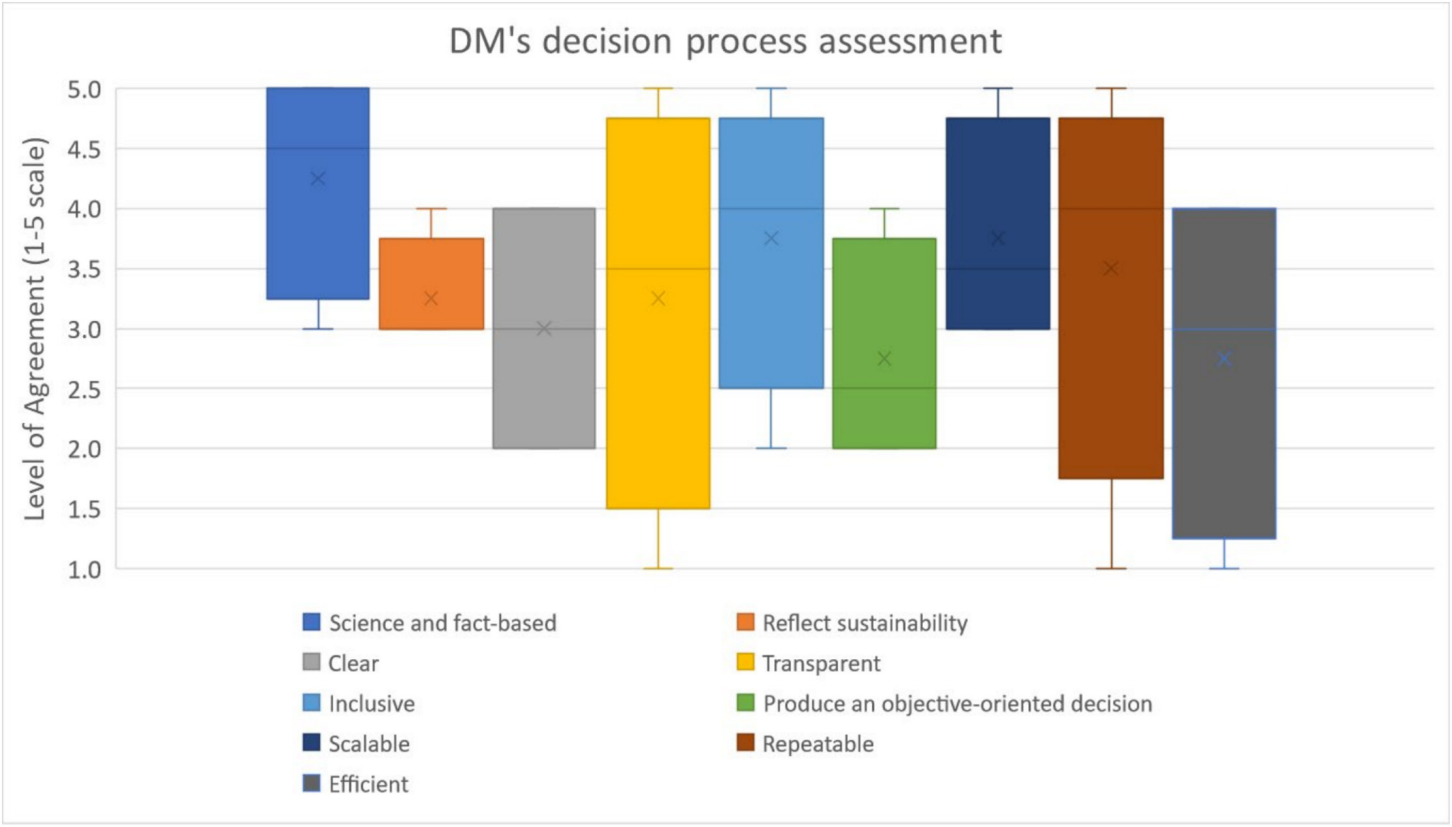
Interviewee assessment of previous decision process (the level of agreement is indicated as follows: 1 = *Strongly Disagree*, 2 = *Disagree*, 3 = *Neither Agree or Disagree*, 4 = *Agree*, 5 = *Strongly Agree*. The mean values are shown as X within the bar).

The interview participants were asked to retrospectively evaluate their participation in the decision process based on the following categories: (1) science and fact‐based, (2) reflect sustainability, (3) clear, (4) transparent, (5) inclusive, (6) produce an objective‐oriented decision, (7) scalable, (8) repeatable, and (9) efficient. The mean value and quartiles of the four respondents interviewed are shown in Figure 12. The existing decision process was considered to be science and fact‐based, inclusive, and scalable, but it was unclear, did not produce an objective‐oriented decision, and was inefficient. The Reflect Sustainability category had low deviation, while the Transparent, Repeatable, and Efficient categories resulted in high deviation. Technical information was considered to be correctly conveyed to the decision‐makers and stakeholders, but the decision process was thought to be unclear and inefficient. Furthermore, the decision process and structure were not clearly understood, nor was the outcome of the final decision. These results indicate that decision‐makers and stakeholders should be given a sufficient explanation of the decision‐making process and structure prior to starting the process.

**FIGURE 13:**
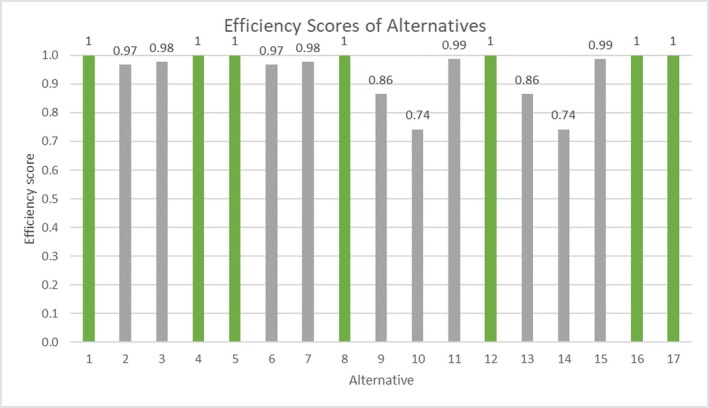
Efficiency scores for alternatives in bar rack and grit chamber decision (green bars represent efficient alternatives (efficiency score = 1) and gray bars represent inefficient alternatives (efficiency score < 1)).

A total of 17 feasible alternatives had been previously developed and analyzed, consisting of four bar screening options coupled with four grit chamber technologies (a total of 16), plus an additional alternative where a newly constructed facility (a combination of fine grit removal and fine screens) would be operated consistently while rehabilitated grit removal units would be used during wet weather. The score for each alternative for the four criteria considered, namely amount of solid removed, construction cost, O&M cost, and social score, was developed by Hazen and WadeTrim (2021). The scores for each alternative are presented in the Supporting Information. The interviewees indicated that the large number of alternatives evaluated made alternative comparison and evaluation challenging. Consequently, we performed a DEA analysis of the 17 alternatives to eliminate inferior solutions and reduce the number before proceeding with the interviews. Construction and O&M costs were selected as input variables, and the amount of solids removed and the social score were the output variables. These selections were made because a small input variable and a large output variable are ideal for the performance of DMUs. Under these conditions, the input‐oriented variable returns to scale BCC model was applied and analyzed. The efficiency score for each alternative was calculated using DEA, as shown in Figure 13. Out of the 17 alternatives, seven (alternatives 1, 4, 5, 8, 12, 16, and 17) had an efficiency score of 1.0 and could be considered efficient. The remaining 10 alternatives had an efficiency score of less than 1.0, were found to be inefficient, and were eliminated from further consideration.

The criteria weights developed for each survey participant using Saaty's scale are presented in Table 2. Participants A, B, and D had a consistency ratio of less than 0.1, indicating consistency in their responses, while the consistency ratio for participant C was greater than 0.1 (duplicate results). Because of this, the final results were developed using the reverse swing method. Overall, the participants indicated a high preference for the amount of SS removed, followed by a moderate preference for the social score and operation cost, and the lowest preference for construction cost. These results suggest that the interviewees perceive that environmental performance and operational feasibility are more valuable than construction cost. Participants A and B showed similar preferences, while participant D showed a stronger preference for operation cost. These differences result in different preferences for individual alternatives.

**TABLE 2:** Criteria weights in the bar rack and grit chamber improvement decision.

Participant	Amount of SS removed	Construction cost	Operation cost	Social score	Consistency ratio
A	58.55%	6.78%	9.79%	24.88%	0.07
B	43.85%	5.43%	15.09%	35.64%	0.08
C	42.95%	20.13%	20.13%	16.78%	—
D	31.61%	4.94%	43.61%	19.83%	0.07
Average	44.24%	9.32%	22.16%	24.28%	—

**FIGURE 14:**
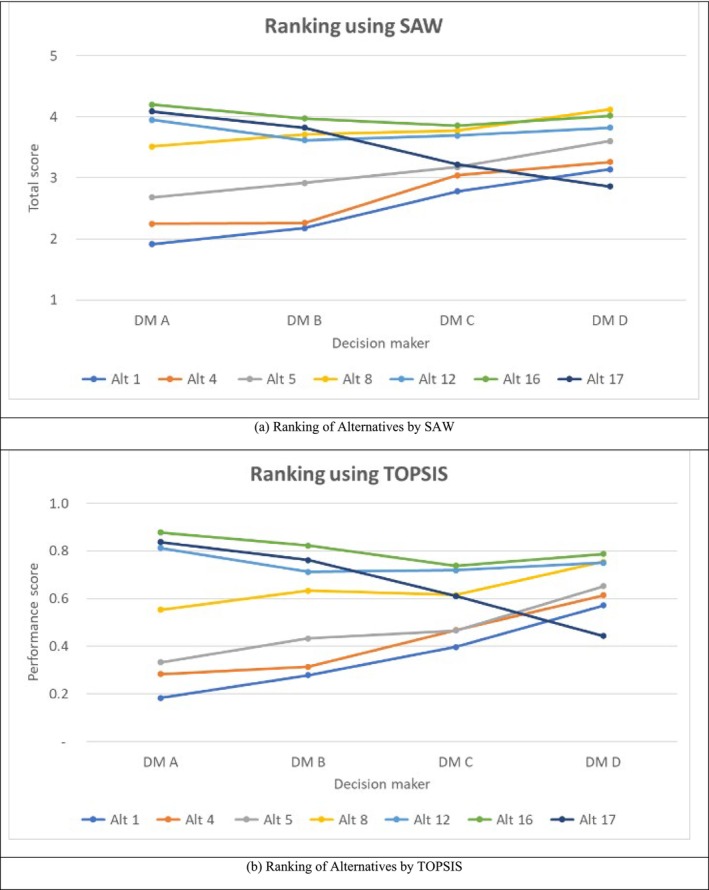
Alternatives ranking by interviewees.

Weights are combined with scores to conduct an MCDA. Utility scales are needed to perform the technical evaluation of the alternatives (USEPA 2015). USEPA (2012). Table 3 presents the utility scales developed for this study. The criteria weights (Table 2) and utility scales (Table 3) were then combined to score each of the seven noninferior alternatives remaining after DEA for each study participant. The results are presented in Figure 14 using the SAW and TOPSIS procedures and indicate similar results for the two procedures. Kalbar et al. (2015) reported that SAW and TOPSIS produced similar results when selecting wastewater treatment alternatives. The results indicated a general preference for alternatives 8, 12, and 16, and a general low preference for alternatives 1, 4, and 5, and mixed results for alternative 17. Different preferences for alternative 17 arise because of different weights for the criteria for the survey participants. Participants A and B had low weights for operation cost (9.79% and 15.09%), while participant D had a very high weight of 43.61% for this criterion.

**TABLE 3:** Utility scale used in this study.

Score	The amount of SS removed	Construction cost	Operation cost	Social score
5	More than 71	Less than 75	Less than 100	More than 66
4	56–70	76–90	101–200	61–65
3	41–55	91–105	201–300	56–60
2	26–40	106–120	301–400	51–55
1	Less than 25	More Than 121	More than 401	Less than 50
Metrics	t/d	$ million	$ thousand/yr	Score

#### 3.2.2 Sensitivity Analysis

Decision‐makers' values are uncertain and cannot be measured precisely, resulting in uncertainty regarding the criteria weights in this study, even though AHP was applied to aid in the objectivity and accuracy of the measurements. Consequently, a sensitivity analysis was performed on the criteria weights of the interviewees. The weight for each of the four criteria was increased separately by 25%, and potential changes in the rankings of each alternative were examined. Given that the total criteria weight must be one, increasing the weight of one criterion required the weight of others to be decreased. The scenarios were constructed as shown in the Supporting Information.

**FIGURE 15:**
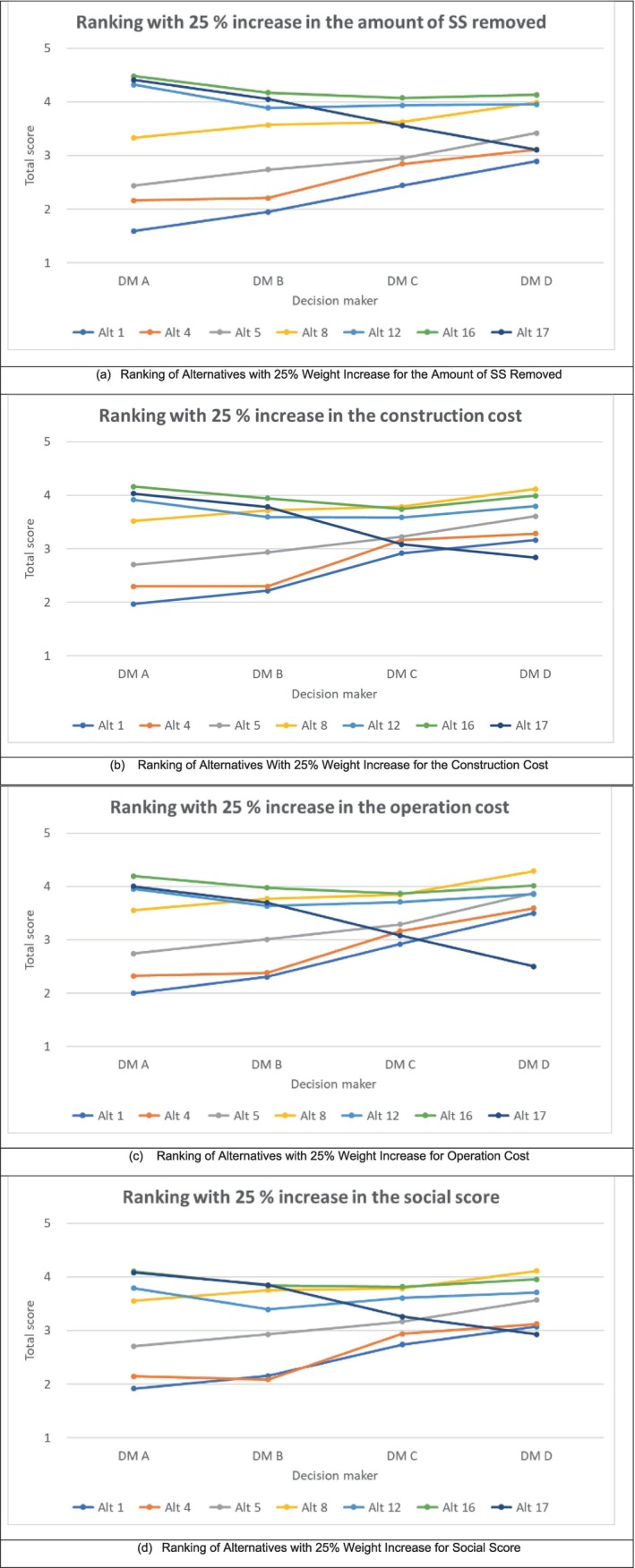
Sensitivity analysis of criteria weights using SAW.

Figure 15 presents the results of the sensitivity analysis in which SAW was applied to each criterion weight to determine rankings. If the amount of the SS removed category increased by 25% (Figure 15a), the rankings of alternatives showed no change, except for alternative 8. Specifically, alternative 8 lowered from 3rd to 4th place for interviewee B, from 2nd to 3rd place for decision‐maker C, and from 1st to 2nd place for interviewee D. If the weight of the construction cost increased by 25% (Figure 15b), changes in ranking did not occur except for interviewee C where the alternatives that were previously ranked 1st and 2nd switched places; alternative 6 rose to 1st place, and alternative 16 fell to 2nd. Also, alternative 17 moved from 4th to 6th place. The rationale for this lack of significant change in interviewees A, B, and D can be attributed to the relatively low weight (4.94%–6.78%) assigned to construction cost. In contrast, interviewee C placed a higher weight of 20.13% on construction cost, which plausibly yielded the observed changes in rankings.

If the weight of operational cost increased by 25% (Figure 15c), minimal change occurred in the rankings of alternatives, except for alternative 17 which dropped from 2nd to 3rd for interviewee B and from 4th to 6th for interviewee C. Finally, if the weight of social score increased by 25% (Figure 15d), there was no reported change for interviewee A, C, and D. For interviewee B, however, alternative 17 emerged as the best alternative, resulting in a proximate total score to alternative 16. In addition, alternative 4 became the worst option for this party. In summary, this study demonstrated that increasing the weight for each criterion by 25% did not result in significant changes in rankings. However, it was found that the change in weight in the amount of SS removed impacted the ranking of alternative 8, and operational cost influenced alternative 17. To further extrapolate, the weight change in the construction cost impacted the rankings for interviewee C, and social cost had an effect on interviewee B.

A sensitivity analysis using TOPSIS as the ranking method was also conducted, with the results presented in the Supporting Information. The results indicated that the ranking of alternatives was less affected by changes in criterion weights when the TOPSIS ranking method was used. An MCDA analysis was also conducted using all of the identified alternatives to determine whether exclusion of the inferior alternatives affected the relative prioritization of alternatives. The results, presented in the Supporting Information, indicate that all inferior options consistently demonstrated lower rankings.

## 4 Discussion

Results from the interviews supporting initial structuring of the Biosolids Master Plan demonstrate how structured interviews can provide useful information to identify relevant parties and to determine decision goals, objectives, and criteria, which are essential components of the initial stage of the decision‐making process presented in Figure 1. Identification of primary responses and keywords for each of the survey questions, followed by an affinity analysis to identify key themes and outcomes of the interviews, provided a useful method for systematic assessment of survey results. Constraints were imposed on this research due to the early stage of Master Plan development, and this work was conducted during COVID‐19, which prevented reaching out to potential external stakeholders, along with the internal stakeholders interviewed. Nevertheless, a range of potential external stakeholders, along with an expanded list of potential internal stakeholders, were identified. Consultation with an expanded group of external and internal stakeholders would be required to further refine the products of these interviews and the synthesis of these results into decision issues, the hierarchy of goals/objectives/criteria, and relevant parties. A similar interview approach could be used to solicit input from this broader range of potential stakeholders.

The preliminary decision hierarchy developed using these interview results included outcomes for goals/objectives, criteria, and constraints. The decision objectives were the triple bottom line of sustainability (environmental, economic, and social) but also included operational feasibility. The inclusion of operational feasibility is indicative of the interests of the GLWA staff interviewed and their priority for ease of operation, reliability, and resilience. Nine decision criteria were proposed based on these objectives. Additionally, technical maturity/reliability and regulatory compliance were suggested as constraints. These criteria illustrate the broader range of considerations required to select implementable wastewater management solutions that can be supported by a broad range of stakeholders.

Results of the retrospective analysis of the bar rack and grit chamber decision process indicated that, while it was science‐ and fact‐based, inclusive, and scalable, improvements were possible to increase clarity, efficiency, and the ability to produce an objective‐oriented outcome. Reduced clarity was attributed at least partially to the large number of alternatives considered. We demonstrated how DEA can be used at the screening and initial evaluation phases, illustrated in Figure 1, to identify inferior alternatives, that is, those alternatives that are systematically inferior to other alternatives under consideration, and to exclude them from further evaluation. This step was well received by the participants in the decision process who were interviewed. DEA has previously been used to compare the performance of existing WWTPs to identify improvement opportunities, as shown in Table 4. In addition, the input variables (e.g., cost) and output variables (e.g., pollutant removal efficiency) of WWTPs change depending on biological treatment technologies such as suspended growth, attached growth, and so on. Molinos‐Senante 2018; Molinos‐Senante et al. 2015; Sala‐Garrido et al. 2011 further used DEA to compare treatment technologies. Here, we used it as a screening technique to eliminate inferior alternatives.

**TABLE 4:** DEA application in wastewater treatment sector in the literature.

References	Methods	DMUs	Input variables	Output variables
Sala‐Garrido et al. (2011)	Output oriented DEA model	99 WWTPs in 4 types (AS, AL, TF, RBC)	Cost	COD removed, nitrogen removed, phosphorus removed
Sala‐Garrido et al. (2012)	DEA model with statistical tolerances	45 WWTPs	Cost	COD removed, SS removed, nitrogen removed
Molinos‐Senante et al. (2015)	MMPI	99 WWTPs in 4 types (AS, AL, TF, RBC)	Cost	COD removed, nitrogen removed, phosphorus removed
Castellet and Molinos‐Senante (2016)	Input‐oriented weighted slacks‐based DEA model	49 WWTPs	Cost (energy, staff, reagents, maintenance, waste management, and other)	COD removed, SS removed, nitrogen removed, phosphorus removed
Molinos‐Senante et al. (2016)	HMPI	204 WWTPs for 2003–2008 period	Cost (energy, staff, reagents, maintenance, waste management, and other)	COD removed, SS removed
Molinos‐Senante (2018)	Metafrontier DEA approach	331 WWTPs in 5 types (CAS, AS with BNPR, AL, TF, RBC)	Energy consumption	BOD removal, SS removal, nitrogen removal, phosphorus removal
Fuentes et al. (2020)	WRDDM model	29 WWTPs	O&M costs and energy consumed	SS removed, COD removed, nitrogen removed, phosphorus removed, sewage sludge produced (undesirable)
Jiang et al. (2020)	Input‐oriented slacked‐based DEA model	861 WWTPs	Operating cost, electricity consumption, and labor	COD removal rate, ammonia nitrogen removal rate, reclaimed water yield, dry sludge yield (undesirable)
Ayyildiz et al. (2021)	Output‐oriented DEA models	30 municipalities	WWTP capacity, daily wastewater amount per person, and number of WWTPs	Amount of wastewater treated, population served by WWTP

Abbreviations: AL, aerated lagoon; AS, activated sludge; BNPR, biological nitrogen and phosphorus removal; BOD, biochemical oxygen demand; CAS, conventional activated sludge; COD, chemical oxygen demand; DEA, data envelopment analysis; DMUs, decision making units; HMPI, Hicks‐Moorsteen productivity index; MMPI, metafrontier Malmquist productivity index; RBC, rotating biological contactor; SS, suspended solids; TF, trickling filter; WRDDM, weighted Russell directional distance model; WWTP, wastewater treatment plant.

MCDA is an effective tool to integrate technical evaluations with the priorities and values of stakeholders and decision‐makers in the decision process. A key outcome indicated by the research presented here is that MCDA can provide a means to identify a modest number of alternatives that can be acceptable (with the top ranking) for a variety of stakeholders, even when their priorities and values differ. MCDA can help decision‐makers and stakeholders understand the similarities and differences in each other's preferences, facilitating further discussion, which promotes collaboration and leads to the transfer of knowledge. Olsson et al. (2011) described this process as social learning. These interactions can enhance knowledge transfer and encourage the discovery of new perspectives on the decision issue. Additionally, there is the opportunity for better alternatives to be developed through the exploration of new alternatives or a combination of existing options. Techniques such as AHP, SAW, and TOPSIS can help facilitate the efficient development of weights (expressions of priorities and values) and present the results of the MCDA in a fashion that is useful for decision‐making.

While this research evaluated two separate decision processes, one in the formulation stage (biosolids management) and the other nearing completion (bar racks and grit chambers), future research should seek to consider decision processes over their entire duration. Such an approach would allow assessment of the impact of various stakeholder engagement strategies on the ultimate outcome of the process. Additional research into successful engagement strategies would also be useful. The results of the bar rack and grit chamber decision process clearly indicate opportunities for improvement. Early and more detailed engagement of stakeholders would have built on the significant operating knowledge of the internal stakeholders to allow for more rapid screening of alternatives, better clarity on the alternatives being evaluated at each stage of the process, and refinement of the evaluation criteria, leading to a more efficient decision process and broader support for the final decision. Simultaneous evaluation of many alternatives can obscure the core decisions, leading to reduced clarity when identifying and making these decisions. More frequent interactions focused on a more limited range of alternatives can stimulate increased social learning, allowing greater consensus and support for the final decision, which enhances implementation. While a more informal interview process is often used in practice, there are benefits to the more formal process used in this research. It allows consistent input by all parties and, consequently, is more transparent and better documented. The affinity analysis allowed the results to be synthesized in a logical and defensible fashion and was not time‐consuming. Overall, results indicated that the overall approach and selective use of the subject tools improved the overall decision process.

In summary, the wastewater management decision‐making process should be comprised of an integrative course of action in which decision‐makers and stakeholders reflect their individual priorities and values in the process, leading to the ultimate decision, as illustrated by the process outlined in Figure 1. The heterogeneous values of the decision‐makers and stakeholders can be identified through decision analysis methodologies, then scrutinized and amalgamated via rigorous debate to attain a final, coherent decision. Such deliberations enhance the mutual understanding of values among decision‐makers and stakeholders, thus invigorating the pursuit of better alternatives. Decision‐making is not a singular, fixed event but an iterative process and a dynamic interchange of knowledge and values, resulting in incremental adjustments. Such a cumulative process ultimately facilitates a communal decision, significantly improving the prospects for successful implementation.

## 5 Summary and Conclusions

In summary, decisions by GLWA concerning biosolids management and bar rack and grit chamber replacement were used to investigate approaches for gaining input to incorporate the priorities and values of decision‐makers and stakeholders into a previously developed wastewater management decision process (Figure 1). The results demonstrated that

1. Structured interviews of potential stakeholders and decision‐makers can produce useful information to identify relevant parties and determine decision goals, objectives, and criteria. Results of such interviews can set the stage for further engagement of relevant stakeholders and decision‐makers in a structured decision process.
2. Identification of primary responses and keywords for each of the survey questions, followed by an affinity analysis to identify key themes and outcomes of the interviews, provided a useful method for systematic assessment of survey results. Techniques such as this should be used to summarize and synthesize interview results in a well‐documented and transparent fashion.
3. An efficient screening stage (see Figure 1) is important to provide clarity on the decisions to be made as the decision process proceeds, resulting in increased efficiency and improved focus on the most viable alternatives. DEA was found in this research to be a useful technique for identifying and removing inferior alternatives.
4. MCDA is an effective tool for integrating the priorities and values of stakeholders and decision‐makers, along with supporting technical evaluations, during both the screening and detailed evaluation of alternatives steps (Figure 1). Interactions between stakeholders and decision‐makers that use MCDA as a framework can enhance knowledge transfer, encourage the discovery of new perspectives, and create the opportunity to develop new alternatives or combinations of existing options.
5. Techniques such as AHP, SAW, and TOPSIS can help facilitate the efficient development of weights (expressions of priorities and values) and present the results of the MCDA in a fashion that is useful for decision‐making.

## Author Contributions

**Daehyun Ko:** funding acquisition, project administration, resources, supervision. **John W. Norton:** conceptualization, funding acquisition, investigation, methodology, resources, validation, writing – review and editing. **Glen T. Daigger:** data curation, software, visualization.

## Conflicts of Interest

The authors declare no conflicts of interest.

## Supporting information


**Figure S1:** Initial Biosolids Decision Evaluation Stakeholder Mapping.
**Figure S2:** Interview result and affinity diagram of decision interests and concerns in GLWA WRRF biosolid management.
**Table S1:** Current issues and keywords at GLWA WRRF biosolid management.
**Table S2:** Emerging issues and keywords at GLWA WRRF biosolid management.
**Table S3:** Decision goals and keywords at GLWA WRRF biosolid management.
**Table S4:** Decision objectives and keywords at GLWA WRRF biosolid management.
**Table S5:** Decision constraints and keywords at GLWA WRRF biosolid management.
**Table S6:** Decision criteria and keywords at GLWA WRRF biosolid management.
**Table S7:** Importance of biosolid decision and keywords at GLWA WRRF biosolid management.
**Table S8:** Roles and responsibilities in GLWA WRRF biosolid management decision.
**Table S9:** Interest and concern of relevant parties and keywords at GLWA WRRF biosolid management.
**Table S10:** Responses and keyword about stakeholders in GLWA WRRF biosolid management decision.
**Table S11:** Responses and keyword about SMEs in GLWA WRRF biosolid management decision.
**Table S12:** Responses and keyword about information required in GLWA WRRF biosolid management decision.
**Table S13:** Responses and keyword about alternatives in GLWA WRRF biosolid management decision.
**Table S14:** Saaty's scale in AHP.
**Table S14:** Generated alternatives and their criteria scores (Hazen & WadeTrim, 2021).
**Table S15:** Generated alternatives and their criteria scores (Hazen & WadeTrim, 2021).
**Table S16:** Alternatives‐criteria matrix of decision‐maker A.
**Table S16:** Alternatives‐criteria matrix of decision‐maker B.
**Table S17:** Alternatives‐criteria matrix of decision‐maker C.
**Table S18:** Alternatives‐criteria matrix of decision‐maker D.
**Figure S3:** Weighted score of decision‐maker A.
**Figure S4:** Weighted score of decision‐maker B.
**Figure S5:** Weighted score of decision‐maker C.
**Figure S6:** Weighted score of decision‐maker D.
**Table S19:** Criteria weights with 25% increase in the amount of SS removed.
**Table S20:** Criteria weights with 25% increase in the construction cost.
**Table S21:** Criteria weights with 25% increase in the operation cost.
**Table S22:** Criteria weights with 25% increase in the social score.
**Figure S7:** Sensitivity analysis of criteria weights using SAW.
**Figure S8:** Sensitivity analysis of criteria weights using TOPSIS.
**Figure S9:** Comparison of alternative rankings with and without DEA screening.

## Data Availability

The data that supports the findings of this study are available in the supplementary material of this article.
