# Supplementary material for: Evaluation of Stakeholder Assessment and Engagement Techniques for Incorporation Into Structured Decision‐Making Processes
Source: Water Environ Res. 2025 Oct 10;97(10):e70187. doi: 10.1002/wer.70187 (PMC12514321; doi:10.1002/wer.70187)
Supplement: Supplementary file 1 — Figure S1: Initial Biosolids Decision Evaluation Stakeholder Mapping. Figure S2: Interview result and affinity diagram of decision interests and concerns in GLWA WRRF biosolid management. Table S1: Current issues and keywords at GLWA WRRF biosolid management. Table S2: Emerging issues and keywords at GLWA WRRF biosolid management. Table S3: Decision goals and keywords at GLWA WRRF biosolid management. Table S4: Decision objectives and keywords at GLWA WRRF biosolid management. Table S5: Decision constraints and keywords at GLWA WRRF biosolid management. Table S6: Decision criteria and keywords at GLWA WRRF biosolid management. Table S7: Importance of biosolid decision and keywords at GLWA WRRF biosolid management. Table S8: Roles and responsibilities in GLWA WRRF biosolid management decision. Table S9: Interest and concern of relevant parties and keywords at GLWA WRRF biosolid management. Table S10: Responses and keyword about stakeholders in GLWA WRRF biosolid management decision. Table S11: Responses and keyword about SMEs in GLWA WRRF biosolid management decision. Table S12: Responses and keyword about information required in GLWA WRRF biosolid management decision. Table S13: Responses and keyword about alternatives in GLWA WRRF biosolid management decision. Table S14: Saaty's scale in AHP. Table S14: Generated alternatives and their criteria scores (Hazen & WadeTrim, 2021). Table S15: Generated alternatives and their criteria scores (Hazen & WadeTrim, 2021). Table S16: Alternatives‐criteria matrix of decision‐maker A. Table S16: Alternatives‐criteria matrix of decision‐maker B. Table S17: Alternatives‐criteria matrix of decision‐maker C. Table S18: Alternatives‐criteria matrix of decision‐maker D. Figure S3: Weighted score of decision‐maker A. Figure S4: Weighted score of decision‐maker B. Figure S5: Weighted score of decision‐maker C. Figure S6: Weighted score of decision‐maker D. Table S19: Criteria weights with 25% increase in the amount of SS removed. Table [file WER-97-e70187-s001.docx]

**SUPPLEMENTAL INFORMATION**

**Incorporating Priorities and Values Into Wastewater Management Decision-Making**

Daehyun Ko^1^, John W. Norton^2^, and Glen T. Daigger^3^

^1^ Ministry of the Environment, Republic of Korea, 11 Doum 6-RO, Sejong-si 30103, Republic of Korea, scruphle@korea.kr
^2^ Great Lakes Water Authority, 735 Randolph, Detroit, Michigan 48226 USA, john.norton@glwater.org
^3^ University of Michigan, 177 EWRE, 1351 Beal Avenue, Ann Arbor, Michigan 48109 USA, [gdaigger@umich.edu](mailto:gdaigger@umich.edu)


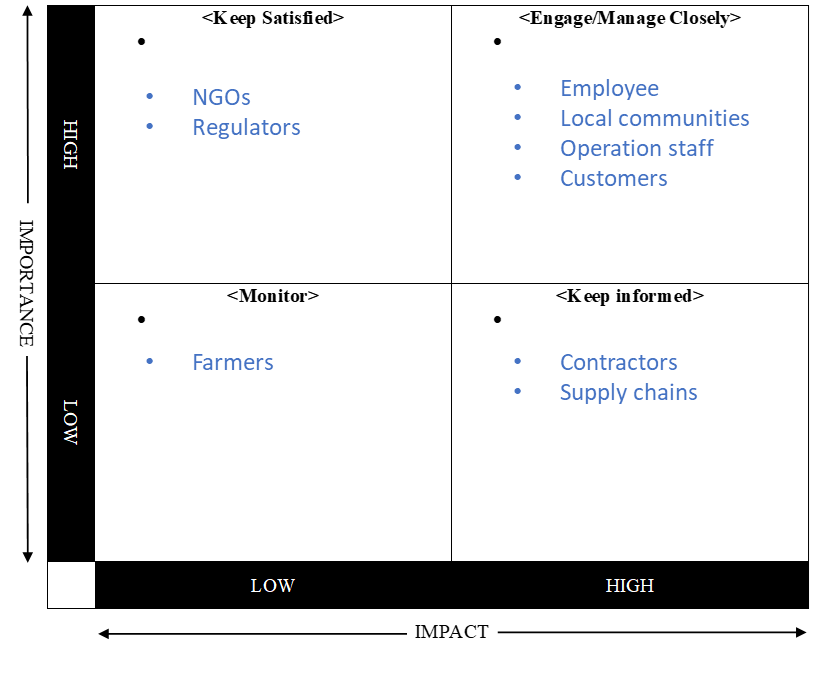


Figure S.1. Initial Biosolids Decision Evaluation Stakeholder Mapping

**Biosolids Management Interview Questions**

**Framing issue**

1. What are the most important problems within the current biosolid management system?
2. What will be the emerging issues to be addressed in the future (20-30 years later)?

Ex) global warming, resource recovery, PFAS, nitrogen control, etc.

1. What is the overarching goal of the decision? What do you want to achieve in the decision?
2. What are the specific objectives of the decision needed to achieve the goal?
3. What are the constraints of the decision?

Ex) Regulation, Financial, physical (land), etc.

**Importance of decision**

1. Why is this decision important to you or your group?
2. What impacts could you anticipate? How much could the decision impact you?
3. What are your roles and responsibilities in the decision?
4. What are your key interests or concerns regarding the decision?

**Identifying relevant parties**

1. Who are the decision-makers in the biosolid management decision?
2. Who are the stakeholders in the biosolid management decision?
   1. Internal stakeholders
   2. External stakeholders
3. Who are the subject matter experts that can participate in the decision?

**Identifying criteria**

1. What are the decision criteria to be considered?

**Possible alternatives**

1. What alternatives (options) could be applied to achieve the identified goal?

**Information**

1. What information is needed to support the decision-making?

| 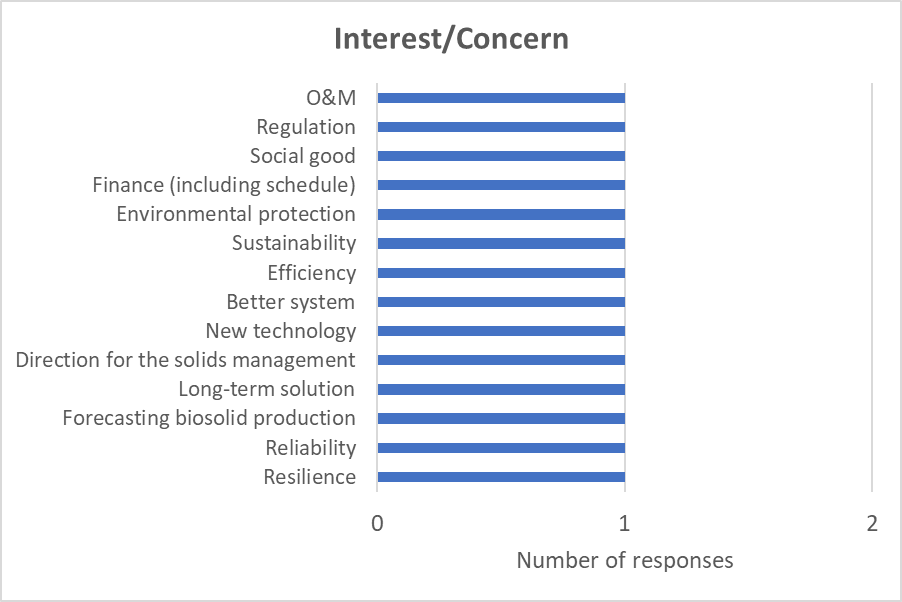 |
| --- |
| 1. Interview response about importance of decision |
| 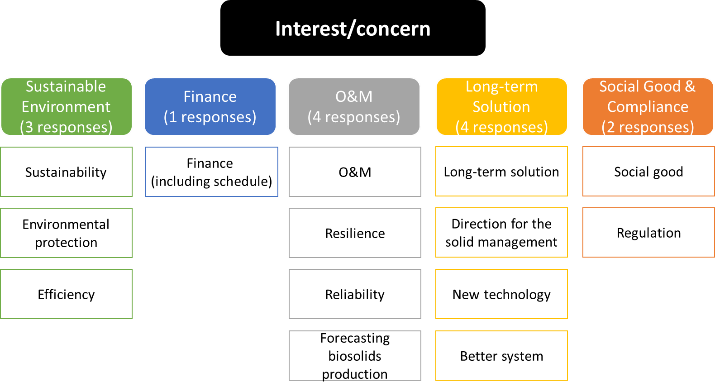 |
| 1. Affinity diagram of importance of decision |

Figure S.2. Interview result and affinity diagram of decision interests and concerns in GLWA WRRF biosolid management

Table S.1. Current issues and keywords at GLWA WRRF biosolid management

| **Current issues** | **Keywords** |
| --- | --- |
| We don't have any issue related to management, but we have some issues in operation and maintenance. Three main issues are cost, maintenance, and logistics gas shut down issue: In two times shut down for a week by gas company. We use a landfill as a process to sludge. landfill is very expensive plus a lot of landfills don't want to take right now because they will have less capacity | Cost,  Maintenance,  Logistics,  Diversification of operation (gas shut down) |
| Forecasting the biosolid production: non-compliance of the NPDES permit (If we recycle the solids for 72 hours, we are in non-compliance of the permit). You need to have a certain level of biosolids inventory so that gravity thickening can happen, and you don't want to have too much of inventory of biosolids because then you have complications of biological phosphorus release recycling. Aging infrastructure: life cycle of complex 2 incinerator will be complete in the next eight years or so. | Forecasting biosolid production,  Aging infrastructure |
| Incineration: demanding process, a potential for a lot of unknowns and risk in air emissions, and financial investment Buffering capabilities: 200/300 - 1200/2000 dry tons a day, inefficient incinerator operation Aged equipment | Incineration risk,  Aging infrastructure |
| Aging equipment: the equipment and the fact that it's end of life | Aging infrastructure |
| What's the best way to deal with biosolids? - Incinerator: not sustainable, air emission, environmental justice issues. - Landfill: wasting the agronomic value of biosolids - Beneficial reuse: toxicity or heavy metal Diversification of operation: we're not going to be able to rely on just one technology, so we're going to have to have a backup, which is probably going to be solidification and landfilling | Incineration risk, Landfill risk,  Resource recovery,  Diversification of operation |
| Not visionary: it's not really looking into the future. Finance looks into the future, it's more dynamic. And I find that that was where the missing link was that, OK, you're telling me what has done. It wasn't visionary and when I think about biosolids or anything environmental, you got to look into the future. You look into the past to see the impact, but you look to the future to see how you're going to manage it | Not visionary |
| the risk associated with having an incinerator system | Incineration risk |

Table S.2. Emerging issues and keywords at GLWA WRRF biosolid management

| **Emerging issues** | **Keywords** |
| --- | --- |
| Cost: landscape, environmental, or regulatory costs will increase. Utility cost also increased significantly  For instance, PFAS related cost increase (land application and landfill will be affected, incinerators have aging issues and don’t have a capacity of 300-400 DTPD in case of limitation of land application and landfill) Aging | Cost,  Micropollutant (PFAS),  Aging infrastructure |
| PFAS: if it has PFAS in it, that's the concern people are reluctant to apply those biosolids if those biosolids containing PFAS, so that is an emerging issue. | Micropollutant (PFAS) |
| Land application: PFAS Maximizing the nutrient extraction: maximize resource extraction and minimize the resource needed to operate is going to be the optimum Workforce: That's a lot more less demanding overall for operational maintenance on day-to-day basis because we should expect that whatever process we select will need less FTEs (Full time equivalents) than the current process. Being able to recruit in that space is going to get harder and harder as time goes by, so more automation. | Micropollutant (PFAS),  Resource recovery,  Workforce |
| Regulatory requirements: PFAS or just whatever's coming out Maintainability: Any type of process, system or equipment that we're putting in so certainly being able to have it sustainable, we want something that we could put in that that works and will work for 20 or 30 years. | Micropollutant (PFAS),  Maintenance |
| Be getting out of the incinerator business Global warming To maximize resource recovery Micropollutants: PFAS or PCBS or DIOXIN'S or whatever else Maximize the beneficial reuse of nitrogen and phosphorus | Global warming,  Micropollutant (PFAS)  Resource recovery  Incineration risk |
| Resource recovery: We're going to have a hard time on recovering the resources that we are currently using up, and I don't think that we are planning enough to be able to deal with it. Cost/aging infrastructure: we've got all this aging infrastructure. And we don't have the money to fix it or the technology you know to make it better or the money to have those the technology makes it better. If you're not looking into the future, you're just looking at how do you deal with things today? | Resource recovery,  Cost,  Aging infrastructure |
| Resource recovery | Resource recovery |
| Global warming: Global warming could be tied to energy consumption which also affects the ratepayers. It's more in the public discourse, Global warming encompasses a number of things to do with energy consumption. Resource recovery: resource recovery affects the bottom line. If we can sell some things, we can offset sort of energy usage and other cost to the customer. The resource recovery embodies the idea of being overall responsible, good stewards Cost: we would like to have a bunch of things right that are doable at the bench scale, which perhaps provide good outputs for us as society, but are so costly that as a society we don't engage in them. | Global warming,  Resource recovery,  Cost |
| Long term sustainability:  - Potentially increased regulation on air pollution  - PFAS related ban on land application. (I would say the most important, the most important issue is long term sustainability of any biosolids management system and our current to you know they both have their individual issues for long term sustainability) | Incineration risk, Micropollutant (PFAS) |

Table S.3. Decision goals and keywords at GLWA WRRF biosolid management

| **Goals** | **Keywords** |
| --- | --- |
| To find out long term biosolids disposal method (To achieve that biosolids are not just destroyed but used as a resource in amending the soil without a concern of the emerging pollutants such as PFAS)  Diversity in the biosolids disposal (drying facility, incinerator, landfill + composting) | Long-term biosolid disposal  Flexibility (diversity) |
| To make a fair equitable fiscally sound and a decision that will be able to support this facility and is in compliance with the regulatory requirements for the next 20 to 30 years. | Sustainability  Compliance  Soundness (fair, equitable, fiscally sound) |
| Working system: I want it to work as anticipated | Feasible system |
| To break it out into different time frames  1. 8-10 years: whenever the end of life is for the incinerators that might limit what we can reasonably do to manage that much sludge within 8 to 10 years  2. long-term goal: a long-term goal depending on what the cost implications are, where it's talked about, biological nutrient removal and sludge digestion and all that stuff. | Feasible system  Long-term biosolid disposal |
| Protecting resources: we make today should be highly protect our resources, protect our environment, protect the land so that down the road we don't even have to think about recovering anything. | Sustainability  Resource recovery |
| More efficient and environmentally friendly solutions | Efficiency  Environmental benefit |
| To provide value to society, and deliver the best triple bottom line for society | Sustainability (for society) |

Table S.4. Decision objectives and keywords at GLWA WRRF biosolid management

| **Objectives** | **Keywords** |
| --- | --- |
| Anaerobic digestion is a good idea because of volume reduction, methane production, and electricity generation, but construction, location, and cost need to be further investigated.  Flexibility: Don't put all eggs in one basket. Keep all three options open and have backup systems. A way to have it run concurrently with the processes that we have right now until we can switch over  Sustainable energy source | Flexibility (diversity)  Sustainable energy source |
| Balance between operability and complexity: We can develop the most complex system that will maximize our organic extractions and nutrient extraction, but it becomes borderline impossible to operate or it requires so much FTE to operate that it becomes like, truly unsustainable  Carbon footprint reduction: we have to look at alternatives there to be able to actually reduce our carbon footprint | Operationability  Resource recovery  Global warming |
| Regulatory compliance (to follow the law)  Cash flow  Operational requirements (to get rid of so much sludge per day, to manage peaks and valleys of the of the sludge production) | Compliance  Cost (cash flow)  Operationability |
| Cost effective solution: Given the CIP (Capital Improvement Plan) budget that I handle | Cost-effectiveness |
| What's good for the environment?  What's good for their ratepayers in terms of cost? | Environmental benefit  Cost (for ratepayer) |
| Regulatory approval: management or disposal is that it's approvable by our regulators because if the regulators don't approve it and we're not gonna get ourselves out of anything, we're just going to spend a bunch of money and still be dealing with an issue.  Operatable and maintainable: the solution at the end in terms of what finally gets constructed has to be simple to operate and simple to maintain if we want to have a chance at success  Long term viability: I would say somehow in the development of a system we need to make sure that we've prospected all of the regulatory authorities, the operations and maintenance and the leadership in an organization to make sure that throwing out on the table everything. That is a potential risk to the project in the future, so that you can try to mitigate those risks now and for the future, if at all possible | Compliance  Operationability  Long-term biosolid disposal |

Table S.5. Decision constraints and keywords at GLWA WRRF biosolid management

| **Constraints** | **Keywords** |
| --- | --- |
| Landfills  Financial cost  Regulatory landscape | Limitation of landfills  Cost  Regulation |
| Financial (cost effective because it's public funds)  Land footprint (physical boundary)  Complication of operability: easy to operate, robust, not too much of a nuisance. | Cost  Land  O&M |
| Operational component: Operational flexibility and ease of operations  Financial | O&M  Cost |
| Financial: we're going into a recession or whatever, money is a big thing  Consensus building: there's a lot of different stakeholders on this, everything from EGLE (Michigan Department of Environment, Great Lakes, and Energy) down to internal GLWA staff, so just coming up with a consensus that everybody can live with | Cost  Consensus building |
| Regulatory acceptance  Operation and maintenance  Physical constraints  Cash flow | Regulation  O&M  Land  Cost |
| Financial: We don't have enough funding even to protect what we have today, let alone what we're going to need in the future.  Political: The political environment does not promote it. We’re not going to want to spend the money. If our governments and our political environment does not think this is important enough for people to invest in, it doesn't provide the incentives or goes the opposite. | Cost  Political environment |
| Regulation  Health and safety  Finance | Regulation  Health and safety  Cost |
| Cost  The ability to do it on the scale that we need to do it | Cost  Scale |
| Regulatory: It has to meet the any constraints of the project have to meet regulatory requirements today and regulatory requirements of the future  Operations and maintenance  Financial: it has to look at the full life cycle cost analysis and it has to make sure that operations and maintenance is possible.  Affordability: If the community can't sustain the debt load to pay for these improvements, whether they're needed or not. how do you make the improvements? The community that you serve can't support that debt load. | Regulation  O&M  Cost  Affordability |

Table S.6. Decision criteria and keywords at GLWA WRRF biosolid management

| **Criteria** | **Keywords** |
| --- | --- |
| Permit  Environmental  Cost  GLWA reputation and public competence: Our relationship with our ratepayers, being able to react quickly and be able to react sustainably (to show the public or our ratepayers that we're moving forward and that we're providing them with a valuable service) | Regulation  Environmental protection  Cost  Public reputation |
| Environmental (conventional and emerging pollutant, including PFAS)  Economic (Cost-effective)  Social: neighborhood concern (smell, noise, truck traffic, health and safety, etc.), team member's health and safety | Environmental protection  Cost  Neighborhood  Health & safety |
| If I prioritize them, it will be economical, environmental, social and recovery | Cost  Environmental protection  Social  Resource recovery |
| Financial impact: How much it's going to cost  Efficiency  Energy and chemicals consumption  Cost of O&M  Volume reduction  Risk factor  Non-cost criteria: ease of operation, risk element, space | Cost  Efficiency  Energy consumption  Volume reduction  Risk  O&M  Space |
| Short term: operational constraints, land constraints, asset condition, regulatory constraints  Long term: sustainability and climate change, beneficial reuse, environmental and social justice | O&M  Space  Asset  Regulation  Sustainability  Global warming  Resource recovery  Environmental justice |
| Impact to the environment  Cost  Feasibility: whatever options we go for has to be feasible and adaptable otherwise obviously it's not going to work.  Required resources: We don't have enough people. Especially after COVID. It's hard to hire people. We've always had a difficult time keeping people. Whatever decision we make, we've got to include what resources this is going to need and whether GLWA has the capacity to absorb those costs, if we need of additional resources | Environmental protection  Cost  Feasibility  Workforce |
| Risk  Cost  Efficiency and resource recovery  Ease of operation  Construction sequence and constructability  Health and safety  Regulatory requirement | Risk  Cost  Efficiency  Resource recovery  O&M  Constructability  Health & safety  Regulation |
| Cost benefit to society  Global warming  Resource recovery | Cost  Global Warming  Resource recovery |
| Regulator's approval  Operations and maintenance  Affordability | Regulation  O&M  Affordability |

Table S.7. Importance of biosolid decision and keywords at GLWA WRRF biosolid management

| **How and why important** | **Keywords** |
| --- | --- |
| Fulfill the permit requirements and public health and safety | Regulation  Health and safety |
| Biosolids management is like mother of all (If you do not manage the biosolids well, It could lead too many non-compliance issues, such as recycling of solids, odor, higher BOD, higher phosphorus pollution, and solids overflowing into the effluent) | Regulation |
| To make sure that we pick the fiscally most responsible project that is sustained for our operations for the next 20-30 years and it provides the best level of service to our communities. | Finance  Sustainability |
| Client: because there's a lot of money on the stake, so I work for our clients, right our customers and so I want to spend their money wisely  Operators: because the operators have a terrible time operating certain equipment now and they're the guys that are in the trench, so realistically, I'm trying to help the guy not use a shovel to pick up sludge off the ground, and so I do my job right then they have a better life.  Everyone: because everybody's got to live with it and ultimately you would like to have a decision that is an amalgamation of everybody's right opinions and, and it may not be the best choice. | Finance  O&M  Consensus |
| Best use of resources: it's just a matter of making sure that we're managing our available resources as best we can, because every dollar that goes to one program, by definition isn't available for other programs. So, it's important to me because we want to make best use of the available resources, so we don't want to spend too much money for something that that isn't going to have benefit to the to the overall organization. | Finance (budget and resource) |
| Future generation: we have future generations that need protection, and the more we recycle and the more things we can take care of our environment today is going to help with the future generation  Pride of our group: The more visionary the biosolids are down the road because I also work at the WRRF. And for my group that takes pride in things like that. GLWA can make things better for the future and make things better for GLWA member partners and communities that serve. So, there's a certain level of pride. | Environmental protection  Pride of group |
| At the end of the day, it's a huge impact on the operations of the organization which trickles into our mission and vision of keeping our communities, keeping things in operation efficiently.  Cost and schedule of me and my group | Social good  Finance |
| My group and me are a subset of society, and so we're trying to do what's right for society.  Financially in terms of a ratepayer and income stream in terms of employability | Social good  Finance |
| Aged incinerators: The existing incineration facility is very old, so maintaining it is a very difficult thing. It requires a substantial amount of money, and it requires a substantial amount of effort on behalf of engineering effort on behalf of operations and maintenance.  Unfavorable work environment of incinerators: The incinerators for our team creates a lot of dust and the environment that you work in in an incinerator building. I would not want my kids to be there and so there's a lot of people that have to be there. But I think it's just generally not the best solution to dispose of solids, and so I guess that's another reason why it's important.  Sustainable solution: Can we do what we're currently doing for disposal of our biosolids, and if it's not, we could soon have only one way to dispose sludge, and that's incineration. And that incineration is, is kind of a House of Cards, needing a lot of work needing a lot of maintenance. Even landfills are becoming places that hold garbage won't even let you dispose of your biosolids anymore because of PFAS issue. | Aging infrastructure  Working environment    Sustainable solution |

Table S.8. Roles and responsibilities in GLWA WRRF biosolid management decision

| **Roles/Responsibilities** | **Expected role** |
| --- | --- |
| Internal stakeholder, SME (Subject Matter Expert) | Internal stakeholder, SME |
| Role: a director of operations for the wastewater  Responsibility: to support my team and have a vision for the success of the wastewater operations group (Just meeting the compliance is a basic responsibility and requirement, but I also need to think in my role to exceed those expectations) | Decision maker |
| My responsibility is to, after taking stakeholders input to potentially be a tiebreaker.  Making sure that the decision is the best for the facility and not necessarily for the short term near term, and it's really the best for the facility for the environment and for the mission of the GLWA. | Decision maker |
| A subject matter expert for electrical and I&C  Facilitation: I try to get all the parties involved. my role typically is reaching out to other individuals, making sure everybody's on the same page trying to start the discussion, and then I'm always the guy that throws out the wacky ideas and sometimes they stick. | Decision maker, SME |
| Interested stakeholder | Internal stakeholder |
| Breaking down decision into feasible components: My role could be really in taking that big picture and breaking it down into pieces and helping people with making their decisions. | Internal stakeholder |
| To evaluate when this project would go in and In which fiscal year we will be able, or fiscal years we will be able to accommodate it? | Internal stakeholder |
| Role: to vocalize, to try and drive what I perceive to a better outcome  Responsibility: Not clear, but I vocalized this that sometimes it's unclear what how the decisions are made in those meetings. I will vocalize my concerns. | Internal stakeholder |
| An executor role so my team executes projects (we were involved in the planning process, but we have a planning team at GLWA, and their intent is to kind of go through a lot of the decisions that we've been made during the planning process. We're not leading that effort, but once those planning decisions have been made right and certain things are established in a master plan, so to speak, you know we're my team is executing those master plan decision) | Decision maker |

Table S.9. Interest and concern of relevant parties and keywords at GLWA WRRF biosolid management

| **Key interests and concerns** | **Keywords** |
| --- | --- |
| Resilience and reliability: when contingency happens, a reliable backup or alternative system is necessary (to keep minimum two option which reliable at different location is much important) | Resilience  Reliability |
| Forecasting model for the biosolids production  The long term this biosolid disposal alternative to find a suitable technology which also by the way, address the PFAS concern. | Forecasting biosolid production  Long-term solution |
| The next steps for the facility and we have to make the decision because we need a direction for the for the solids management prior to air emission compliance issues. | Direction for the solids management |
| New technology  Better system: I also do want to make the plant a better place. And I want to make operations easier, so it's getting a system that is better than what we have. | New technology  Better system |
| Make efficient use of our available resources  Sustainability: global warming, climate change, environmental justice | Efficiency  Sustainability |
| It's not going to be given the importance that it deserves. As per usual, our environment always comes secondary. It's always like, oh, that's not going to happen or that's down the road. We can deal with it later. | Environmental protection |
| To stay within schedule and on budget: When will this project take place? What will it cost and within what time, what framework? What will the impact from a perspective of other things that operate like from an operation standpoint? What do you need to do in the interim, and what type of projects will take place? To in the interim as a predecessor project or as a successor project, all of the complex cycle of running that project into when you proceed into implementation. | Finance  Schedule |
| Interests: a good outcome for society  Concerns: Get the decision that wouldn't good for society overall | Social good |
| Regulatory requirements, operations, and maintenance. | Regulation  O&M |

Table S.10. Responses and keyword about stakeholders in GLWA WRRF biosolid management decision

| **Stakeholders** | **Keyword**  **(Internal stakeholders)** | **Keyword**  **(External stakeholders)** |
| --- | --- | --- |
| Ratepayers (depend on the cost)  Financial system in GLWA  Engineering  GLWA management  Experts | Finance  Engineering | Ratepayers  Experts |
| Internal: operation, maintenance  External: member partner communities, private partners (NEFCO), regulatory, neighborhood | O&M | Member partners  Private partners (NEFCO)  Regulatory  Neighborhood |
| Internal: operation/maintenance  External: EGLE, member partners | O&M | Regulatory  Member partners |
| Internal: energy, CIP team (financing), Research innovation group, plant O&M,  External: EGLE, NEFCO, waste management and other disposal companies | Energy  Finance  Research innovation  O&M | Regulatory  Private partners (NEFCO, waste management and other disposal companies) |
| Internal: engineering, maintenance, operation, legal, finance  External: EGLE (air quality, NPDES, waste management), community groups (southeast or southwest Michigan), surrounding neighbors | Engineering  O&M  Legal team  Finance | Regulatory  Communities  Neighborhood |
| Internal: asset management team, outreach group  External: member partners, communities | Finance  Outreach group | Member partners  Communities |
| Internal: managed operation, maintenance, engineer, finance, managed implementation, implementation of the design and construction, and then the operation of the solution  External: member partners, board of directors | O&M  Engineering  Finance  Implementation  Design  Construction | Member partners  Board of directors |
| Internal: operation and maintenance, engineers and all of those other support staff  External: academia, government regulators, community stakeholders | O&M  Engineering  Support staff | Academia  Regulators  Communities |

* NEFCO (New England Fertilizer Company). EGLE (Michigan Department of Environment, Great Lakes, and Energy), NPDES (National Pollutant Discharge Elimination System)

Table S.11. Responses and keyword about SMEs in GLWA WRRF biosolid management decision

| **SMEs** | **Keywords** |
| --- | --- |
| Consulting communities  University partners  The other national utilities that have implemented some of these decision | Consulting  University  Other national utilities |
| Internal experts: research and innovation, electrical and I&C (Instrumentation and Control), process, structure, civil | Internal experts |
| The external gurus who know biosolids in and out  The people who will operate the system, so the feedback from those team members can play a huge role (a lot of times, people, the those who operate the system are forgotten until you have a design and it's done and now you present it to them and they're like, well this is not gonna work simply because you know of this and that, or the sequence of construction or so, bringing the people who operate the system in on the decision from early on really helps because they will tell you the lessons learned, what works, what doesn't, what to be aware of) | External experts  Internal experts (operation) |

Table S.12. Responses and keyword about information required in GLWA WRRF biosolid management decision

| **Required information** | **Keyword** |
| --- | --- |
| How robust are and how proven that technology is?  The effectiveness, the efficiency and the easy operability of the technology  Finance | Technical maturity  Efficiency  O&M  Cost |
| Costs of construction  Costs of operation and maintenance  Understanding of resources: FTEs (Full-Time Equivalents) necessary to operate the facility  The operational flexibility within the alternatives  Modularity: long term expansion of the process, if regulatory requirements are brought forth at the end of the day, we still have flexibility to be able to utilize what we invested in, but also add additional processes to be able to achieve | Cost  Workforce  Flexibility  Modularity |
| Information related to the criteria: volume reduction, safety (pressure and temperature), operation (batch or continuous), technical maturity, capacity (How that gets upsized for our flows), financial information (O&M cost, energy and chemicals cost, revenue), understanding of the risk, operationability (at low flows and high flows during dry weather and wet weather) | Volume reduction  Safety  O&M  Technical maturity  Capacity  Cost  Risk |
| Goals  Costs  Resource required  Timeline  Ultimate benefit  Cost benefit analysis | Goals  Cost  Resource required  Timeline  Benefit  Cost/benefit analysis |
| Existing cost: labor costs, maintenance costs, operational costs, energy costs, chemical costs  Existing capabilities or capacities: how to run an incinerator, how to landfill, how to dry sludge  Technical maturity: what's available in terms of expertise to support whatever alternative ends up being selected  Neighborhood opinion: whatever the constraints are in terms of the neighborhood setbacks  Regulation compliance: whatever the city of Detroit codes are  Stakeholders’ opinion: whatever Southwest Detroit stakeholder environmental stakeholders think is  Public stakeholder's opinion  EGLE's prediction: we need to also check in with EGLE’s crystal ball on waste management, air and NPDES and just make sure that we're not missing something to do an analysis and get their crystal ball out there for where they see things going | Cost  Capacity  Technical maturity  Neighborhood opinion  Regulation  Stakeholder opinion  Public opinion |
| An alternative with a defined scope with boundary conditions defined as well constraints  Opportunities  Schedule and implementation  Feasibility analysis (Present worth analysis for each alternative) | Alternatives  Opportunities  Timeline  Feasibility |
| Economic or cost information  Science information: information about the real world, operability, and feasibility of the systems  Environmental information: information related to how it affects the environment to get back to those things about global climate warming and stakeholders  Resource recovery possibilities: beneficial reuse of biosolid  Regulatory position or support: If you have a great technology but the regulator is not going to support it right, that needs to be brought in. You don't want to be spend a bunch of time and money to make a decision that's not feasible | Cost  O&M  Feasibility  Environmental impact  Resource recovery  Regulation |
| Life cycle cost  Number of assets  The level of maintenance: Is it one hour a week or is it 40 hours a week? Does it take two guys? Does it take 10 guys?  Effectiveness and reliability  Resilience  Previous studies: What research has been done on it and has it actually been implemented in real life? And how long has it been implemented? What were the results of previous projects? how much did they cost? What were lessons learned? Did it ultimately meet the regulatory requirements? There's just all sorts of information on other installations on other operations and maintenance experience. | Cost  Asset  O&M  Efficiency  Resilience  Previous studies |

Table S.13. Responses and keyword about alternatives in GLWA WRRF biosolid management decision

| **Alternatives** | **Keyword** |
| --- | --- |
| Wind and solar: reduce the costs of operating a biosolids facility, generating own power  Alternative fuel sources for doing biosolids  Technologies with capacity and reliability (flexible and could meet maximum capacity) | Renewable energy, Reliable technologies |
| Anaerobic digestion (AD)  HTL  Thermal hydrolysis process (THP)  Composting  Pyrolysis | AD  HTL  THP  Composting  Pyrolysis |
| Anaerobic digestion, thermal hydrolysis, we need that for footprint management. I think anaerobic digestion is important.  The entire flow scheme versus partial load scheme: Optimizing, maximizing the most nutritious product to be hydrolyzed like digested versus having our entire flow stream digested  Utilization of existing assets: maximizing our existing assets. In repurposing and optimizing our dryer facility, that facility we have an existing contract and then we will have another and at the end of the contract obligation is that they will reinvest in the facility, and they restore it back to the useful life of the facility. | AD  Partial load scheme  Utilization of existing asset |
| Separation: We would probably not be treating our complete biosolid stream. We're going to take a side stream and we're gonna do some of it, and then some of it's going to do something else. What part of the stream are we going to treat with this, or what part of the stream are we going to treat with that?  Method of construction | Partial load scheme  Method of construction |

**Bar Rack and Grit Chamber Interview Questions**

**Background question**

1. Why did you (or what led you to) participate in the decision?
2. What was your role and responsibilities in the decision?
3. What were your key interests or concerns pertaining to the decision?
   1. Why are these interests or concerns important to you or your group?

**Framing issue**

1. In your opinion, what was the problem with the existing facilities?
2. What was the goal of the decision?
   1. Were you well aware of the goal throughout the process?
   2. Did you agree with the goal of the decision?
      1. Why or why not?
      2. If not, what did you think the goal should be?
3. What were the objectives of the decision needed to achieve the goal?
   1. Were you well aware of the objectives throughout the process?
   2. Did you agree with the objectives of the decision?
      1. Why or why not?
      2. If not, what did you think the objectives should be?
4. In your opinion, what were the constraints of the decision?

**Identifying criteria**

1. What were the decision criteria?
   1. Were you well aware of the decision criteria throughout the decision process?
   2. How much did the criteria align with the goal/objectives of the decision?
2. Were you satisfied with the criteria?
   1. If you were satisfied with the criteria, why or what attributes contributed to this?
   2. If you were neutral or not satisfied with the criteria, what criteria did you think should be included or excluded?

**Weighting criteria**

1. Did you participate in the weighting process (preference of the criteria)?
   1. If you participated in the weighting, what was your preference?
      1. What were the most important criteria?
   2. If you did not participate in the weighting, what is your preference?
2. This question uses AHP (Analytic Hierarchy Process) to analyze the decision’s criteria weighting. Please use the following scale to express your preference between the two criteria.

**Final decision and relevant parties**

1. What was the final decision?
2. Do you support the final decision?
   1. If not, why do you not support the decision?
3. What was the major impact of the decision?
   1. Why is the impact important?
   2. How much would the decision impact you?
4. What happened in the decision?
   1. What was successful with the decision?
   2. What weaknesses, or areas for improvement, existed around the decision?
   3. What can be done differently in the decision-making process?
5. Who were the relevant parties (decision-makers/stakeholders/experts) that should have been engaged in the decision but were not? (Inversely, were there any parties involved that should not have been?)

**Decision characteristics**

Based on a 1-5 scale, 1 for the least agreeable and 5 for the most agreeable.

For each of the below statements, please rate your level of agreement from 1 to 5, where 1 = Strongly Disagree • 2 = Disagree • 3 = Neither Agree or Disagree • 4 = Agree • 5 = Strongly Agree

1. The decision process was science and fact-based.
   - Science and fact-based: Relevant information that the decision should be based on is incorporated, and extraneous information is not given undue weight. The methodologies in the decision-making process utilize the up-to-date scientific process and knowledge.
2. The decision process reflected sustainability.
   - Reflects sustainability: The decision process should reflect a variety of relevant perspectives affecting the decision, supporting sustainable outcomes. The triple bottom line of sustainability is sufficiently achieved through the decision-making process. The environmental, economic, and social perspectives are considered in the process.
3. The decision process was clear.
   - Clear: The decision to be made is clearly stated. We may require the analysis of alternatives as a means to assist with decision-making, but the core decision to be made is clearly stated.
4. The decision process was transparent.
   - Transparent: The process is clearly defined and documented.
5. The decision process was inclusive.
   - Inclusive: The roles and responsibilities of the relevant parties (decision-makers and stakeholders) are well defined, with the participation of the relevant parties appropriate to their identified role
6. The decision process produced an objective-oriented decision.
   - Produces an objective-oriented decision: The decision process can support the achievement of the objectives of the decision, which the relevant parties support.
7. The decision process was scalable.
   - Scalable: The components of the decision process are adjustable to the nature and scope of the decision to be made.
8. The decision process was repeatable.
   - Repeatable: The components of the process are clearly defined.
9. The decision process was efficient.
   - Efficient: Support, such as tools, checklists, and defined procedures, is provided to accomplish the necessary work as easily as possible.

**Paired comparison in AHP**

This question uses AHP (Analytic Hierarchy Process) to analyze the decision criteria weighting. Please use the following scale to express your preference between the two criteria.

Table S.14. Saaty's scale in AHP

| Intensity of importance | Definition |
| --- | --- |
| 1 | Equal importance |
| 3 | Moderate importance |
| 5 | Essential or strong importance |
| 7 | Very strong importance |
| 9 | Extreme importance |
| 2,4,6,8 | Intermediate values between the two adjacent judgements |

- 1. How much more is “The amount of SS removed” preferred over “Operation cost”?

| The amount of SS removed | | |  |  |  |  | Operation cost | |
| --- | --- | --- | --- | --- | --- | --- | --- | --- |
|  |  |  |  |  |  |  |  |  |
| 9 | 7 | 5 | 3 | 1 | 3 | 5 | 7 | 9 |

- 1. How much more is “Operation cost” preferred over “Construction cost”?

| Operation cost | |  |  |  |  |  | Construction cost | |
| --- | --- | --- | --- | --- | --- | --- | --- | --- |
|  |  |  |  |  |  |  |  |  |
| 9 | 7 | 5 | 3 | 1 | 3 | 5 | 7 | 9 |

- 1. How much more is “The amount of SS removed” preferred over “Construction cost”?

| The amount of SS removed | | |  |  |  |  | Construction cost | |
| --- | --- | --- | --- | --- | --- | --- | --- | --- |
|  |  |  |  |  |  |  |  |  |
| 9 | 7 | 5 | 3 | 1 | 3 | 5 | 7 | 9 |

- 1. How much more is “The amount of SS removed” preferred over “Social score”?

| The amount of SS removed | | |  |  |  |  | Social score | |
| --- | --- | --- | --- | --- | --- | --- | --- | --- |
|  |  |  |  |  |  |  |  |  |
| 9 | 7 | 5 | 3 | 1 | 3 | 5 | 7 | 9 |

- 1. How much more is “Operation cost” preferred over “Social score”?

| Operation cost | |  |  |  |  |  | Social score | |
| --- | --- | --- | --- | --- | --- | --- | --- | --- |
|  |  |  |  |  |  |  |  |  |
| 9 | 7 | 5 | 3 | 1 | 3 | 5 | 7 | 9 |

- 1. How much more is “Construction cost” preferred over “Social score”?

| Construction cost | |  |  |  |  |  | Social score | |
| --- | --- | --- | --- | --- | --- | --- | --- | --- |
|  |  |  |  |  |  |  |  |  |
| 9 | 7 | 5 | 3 | 1 | 3 | 5 | 7 | 9 |
|  |  |  |  |  |  |  |  |  |

Table S.14. Generated alternatives and their criteria scores (Hazen & WadeTrim, 2021)

| **Alternatives** | **Bar Screening** | **Grit Chamber** | **The amount of SS removed (t/d)** | **Construction cost ($M)** | **O&M cost ($/yr)** | **Social score** |
| --- | --- | --- | --- | --- | --- | --- |
| 1 | A | A | 19 | 65 | 60,820 | 53 |
| 2 | A | B | 19 | 81 | 62,874 | 53 |
| 3 | A | C | 37 | 93 | 62,271 | 43 |
| 4 | A | D | 37 | 71 | 60,820 | 49 |
| 5 | B | A | 31 | 76 | 60,820 | 57 |
| 6 | B | B | 31 | 92 | 62,874 | 57 |
| 7 | B | C | 49 | 104 | 62,271 | 52 |
| 8 | B | D | 49 | 81 | 60,820 | 61 |
| 9 | C | A | 52 | 97 | 116,391 | 44 |
| 10 | C | B | 52 | 113 | 118,445 | 44 |
| 11 | C | C | 75 | 125 | 117,842 | 40 |
| 12 | C | D | 75 | 106 | 116,391 | 51 |
| 13 | D | A | 52 | 97 | 116,391 | 47 |
| 14 | D | B | 52 | 113 | 118,445 | 47 |
| 15 | D | C | 75 | 125 | 117,842 | 47 |
| 16 | D | D | 75 | 106 | 116,391 | 56 |
| 17 | Separate dry weather | | 75 | 130 | 462,669 | 62 |

* Bar screening: A (replacing the existing screens (0.75 inch) with smaller opening coarse screens (0.50 inch) in the existing channels), B (replacing the existing screens (0.75 inch) with 0.25 inch bar screens and construction of additional screen channels), C (replacing the existing screens with 0.5 inch coarse screens and adding fine screens within the grit chamber inlet), D (replacing the existing screens with 0.5 inch coarse screens and adding fine screens within the grit chamber outlet).

** Grit chamber: A (rehabilitating the existing aerated grit chambers with screw conveyors), B (rehabilitating the existing aerated grit chambers with submersible grit pump), C (stacked tray grit removal units), D (stirred vortex grit removal units),

*** Separate dry weather: a newly constructed facility (finer grit removal and fine screens) to improve performance during dry weather while maintaining the peak capacity during wet weather.

**Alternatives-criteria matrix of decision-makers**

Table A.15. Generated alternatives and their criteria scores (Hazen & WadeTrim, 2021)

| **Alternatives** | **Bar Screening** | **Grit Chamber** | **The amount of SS removed (t/d)** | **Construction cost ($M)** | **O&M cost ($/yr)** | **Social score** |
| --- | --- | --- | --- | --- | --- | --- |
| 1 | A | A | 19 | 65 | 60,820 | 53 |
| 2 | A | B | 19 | 81 | 62,874 | 53 |
| 3 | A | C | 37 | 93 | 62,271 | 43 |
| 4 | A | D | 37 | 71 | 60,820 | 49 |
| 5 | B | A | 31 | 76 | 60,820 | 57 |
| 6 | B | B | 31 | 92 | 62,874 | 57 |
| 7 | B | C | 49 | 104 | 62,271 | 52 |
| 8 | B | D | 49 | 81 | 60,820 | 61 |
| 9 | C | A | 52 | 97 | 116,391 | 44 |
| 10 | C | B | 52 | 113 | 118,445 | 44 |
| 11 | C | C | 75 | 125 | 117,842 | 40 |
| 12 | C | D | 75 | 106 | 116,391 | 51 |
| 13 | D | A | 52 | 97 | 116,391 | 47 |
| 14 | D | B | 52 | 113 | 118,445 | 47 |
| 15 | D | C | 75 | 125 | 117,842 | 47 |
| 16 | D | D | 75 | 106 | 116,391 | 56 |
| 17 | Separate dry weather | | 75 | 130 | 462,669 | 62 |

* Bar screening: A (replacing the existing screens (0.75 inch) with smaller opening coarse screens (0.50 inch) in the existing channels), B (replacing the existing screens (0.75 inch) with 0.25 inch bar screens and construction of additional screen channels), C (replacing the existing screens with 0.5 inch coarse screens and adding fine screens within the grit chamber inlet), D (replacing the existing screens with 0.5 inch coarse screens and adding fine screens within the grit chamber outlet).

** Grit chamber: A (rehabilitating the existing aerated grit chambers with screw conveyors), B (rehabilitating the existing aerated grit chambers with submersible grit pump), C (stacked tray grit removal units), D (stirred vortex grit removal units),

*** Separate dry weather: a newly constructed facility (finer grit removal and fine screens) to improve performance during dry weather while maintaining the peak capacity during wet weather.

Table S.16. Alternatives-criteria matrix of decision-maker A

| **Decision-maker A** | **Weight** | **Alt 1** | **Alt 4** | **Alt 5** | **Alt 8** | **Alt 12** | **Alt 16** | **Alt 17** |
| --- | --- | --- | --- | --- | --- | --- | --- | --- |
| Amount of SS removed | 58.55% | 1 | 2 | 2 | 3 | 5 | 5 | 5 |
| Construction cost | 6.78% | 5 | 5 | 4 | 4 | 2 | 2 | 1 |
| Operation cost | 9.79% | 5 | 5 | 5 | 5 | 4 | 4 | 1 |
| Social score | 24.88% | 2 | 1 | 3 | 4 | 2 | 3 | 4 |

Table S.16. Alternatives-criteria matrix of decision-maker B

| **Decision-maker B** | **Weight** | **Alt 1** | **Alt 4** | **Alt 5** | **Alt 8** | **Alt 12** | **Alt 16** | **Alt 17** |
| --- | --- | --- | --- | --- | --- | --- | --- | --- |
| Amount of SS removed | 43.85% | 1 | 2 | 2 | 3 | 5 | 5 | 5 |
| Construction cost | 5.43% | 5 | 5 | 4 | 4 | 2 | 2 | 1 |
| Operation cost | 15.09% | 5 | 5 | 5 | 5 | 4 | 4 | 1 |
| Social score | 35.64% | 2 | 1 | 3 | 4 | 2 | 3 | 4 |

Table S.17. Alternatives-criteria matrix of decision-maker C

| **Decision-maker C** | **Weight** | **Alt 1** | **Alt 4** | **Alt 5** | **Alt 8** | **Alt 12** | **Alt 16** | **Alt 17** |
| --- | --- | --- | --- | --- | --- | --- | --- | --- |
| Amount of SS removed | 42.95% | 1 | 2 | 2 | 3 | 5 | 5 | 5 |
| Construction cost | 20.13% | 5 | 5 | 4 | 4 | 2 | 2 | 1 |
| Operation cost | 20.13% | 5 | 5 | 5 | 5 | 4 | 4 | 1 |
| Social score | 16.78% | 2 | 1 | 3 | 4 | 2 | 3 | 4 |

Table S.18. Alternatives-criteria matrix of decision-maker D

| **Decision-maker D** | **Weight** | **Alt 1** | **Alt 4** | **Alt 5** | **Alt 8** | **Alt 12** | **Alt 16** | **Alt 17** |
| --- | --- | --- | --- | --- | --- | --- | --- | --- |
| Amount of SS removed | 31.61% | 1 | 2 | 2 | 3 | 5 | 5 | 5 |
| Construction cost | 4.94% | 5 | 5 | 4 | 4 | 2 | 2 | 1 |
| Operation cost | 43.61% | 5 | 5 | 5 | 5 | 4 | 4 | 1 |
| Social score | 19.83% | 2 | 1 | 3 | 4 | 2 | 3 | 4 |

**Weighted score of each decision-maker**


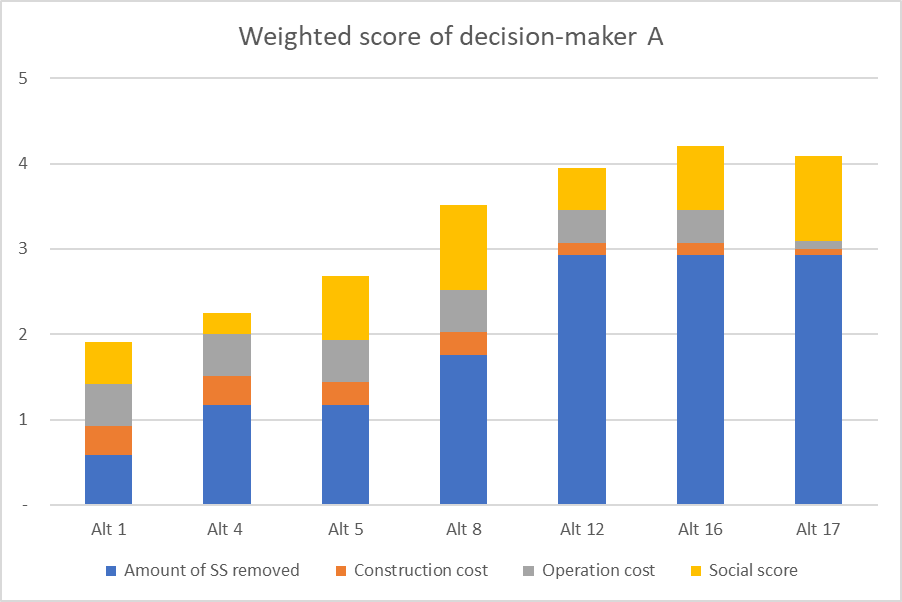


Figure S.3. Weighted score of decision-maker A


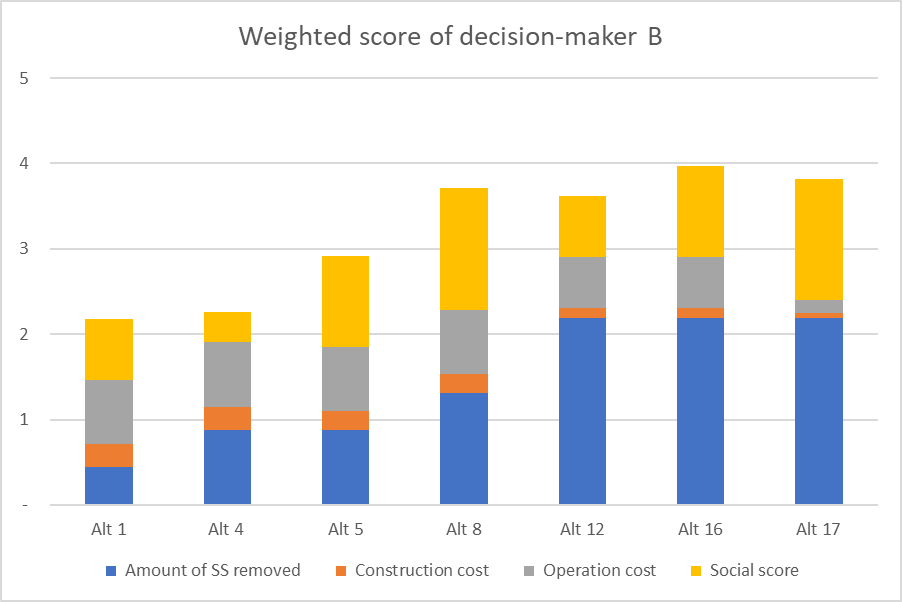


Figure S.4. Weighted score of decision-maker B


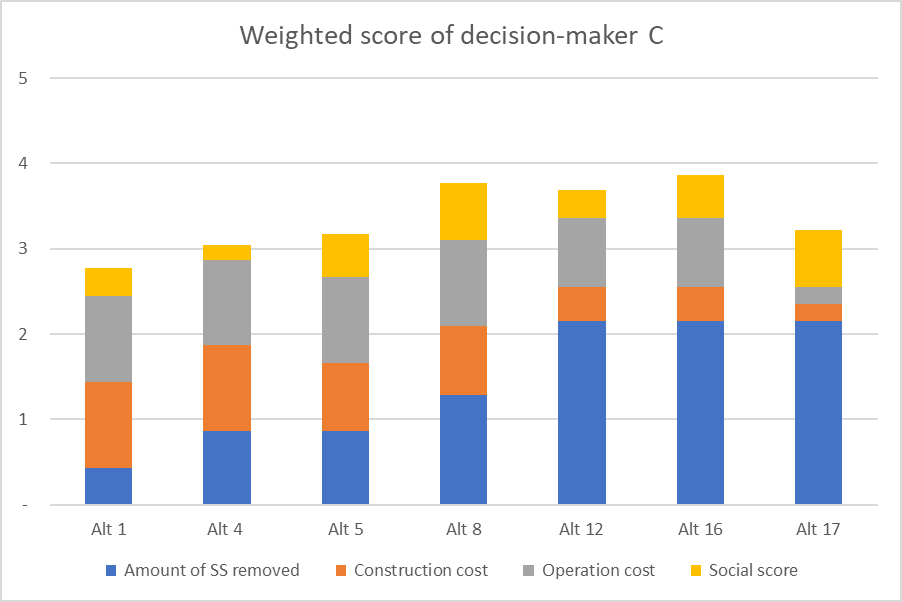


Figure S.5. Weighted score of decision-maker C


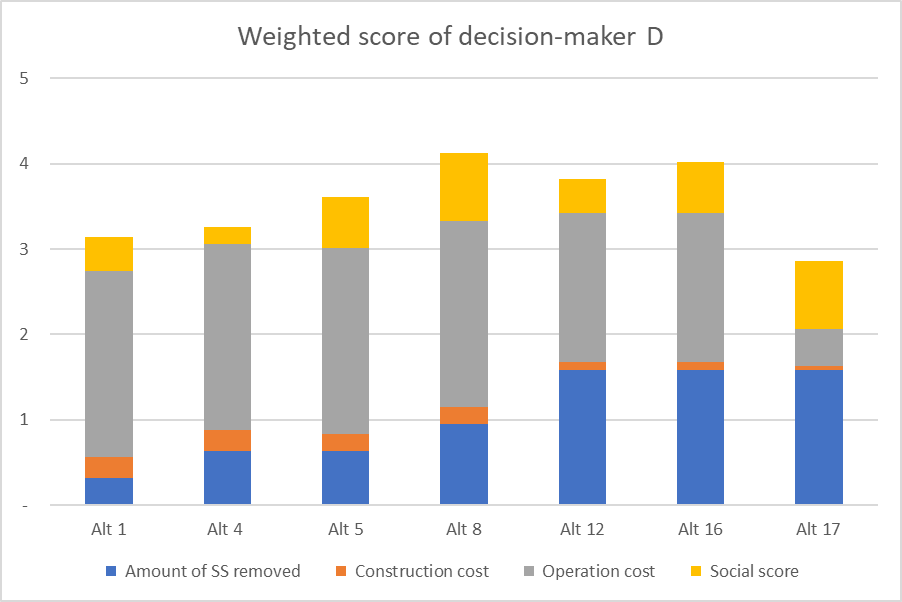


Figure S.6. Weighted score of decision-maker D

**Criteria weights simulated in the sensitivity analysis.**

Table S.19. Criteria weights with 25% increase in the amount of SS removed

| **Criteria** | **Decision-maker A** | **Decision-maker B** | **Decision-maker C** | **Decision-maker D** |
| --- | --- | --- | --- | --- |
| The amount of SS removed | 73.19% | 54.81% | 53.69% | 39.52% |
| Construction cost | 4.38% | 4.37% | 16.34% | 4.37% |
| Operation cost | 6.33% | 12.14% | 16.34% | 38.57% |
| Social score | 16.09% | 28.68% | 13.62% | 17.54% |

Table S.20. Criteria weights with 25% increase in the construction cost

| **Criteria** | **Decision-maker A** | **Decision-maker B** | **Decision-maker C** | **Decision-maker D** |
| --- | --- | --- | --- | --- |
| The amount of SS removed | 57.49% | 43.22% | 40.25% | 31.20% |
| Construction cost | 8.47% | 6.79% | 25.17% | 6.18% |
| Operation cost | 9.61% | 14.87% | 18.87% | 43.05% |
| Social score | 24.43% | 35.12% | 15.72% | 19.57% |

Table S.21. Criteria weights with 25% increase in the operation cost

| **Criteria** | **Decision-maker A** | **Decision-maker B** | **Decision-maker C** | **Decision-maker D** |
| --- | --- | --- | --- | --- |
| The amount of SS removed | 56.96% | 41.90% | 40.25% | 25.50% |
| Construction cost | 6.59% | 5.19% | 18.87% | 3.99% |
| Operation cost | 12.24% | 18.86% | 25.17% | 54.52% |
| Social score | 24.21% | 34.05% | 15.72% | 16.00% |

Table S.22. Criteria weights with 25% increase in the social score

| **Criteria** | **Decision-maker A** | **Decision-maker B** | **Decision-maker C** | **Decision-maker D** |
| --- | --- | --- | --- | --- |
| The amount of SS removed | 53.70% | 37.78% | 40.79% | 29.66% |
| Construction cost | 6.22% | 4.68% | 19.12% | 4.64% |
| Operation cost | 8.98% | 13.00% | 19.12% | 40.92% |
| Social score | 31.10% | 44.54% | 20.97% | 24.79% |

### **Sensitivity Analysis**

Decision-makers’ values are highly uncertain and cannot be measured precisely. There was still a high level of uncertainty regarding the criteria weights in this study, although AHP was applied to aid in the objectivity and accuracy of the measurements. To address this variability, sensitivity analysis was performed on the criteria weights of the decision-makers. If the four criteria weights increased by 25%, the changes in the rankings of each alternative were examined. Given the total criteria weight must be one, one criteria weight’s increase would correspondingly decrease the others. The scenarios were constructed as shown in Appendix D.

The results in Figure S.7 depict the sensitivity analysis in which SAW was applied to each criteria weight to determine rankings. If the amount of SS removed category increased by 25%

|  |  |
| --- | --- |
| 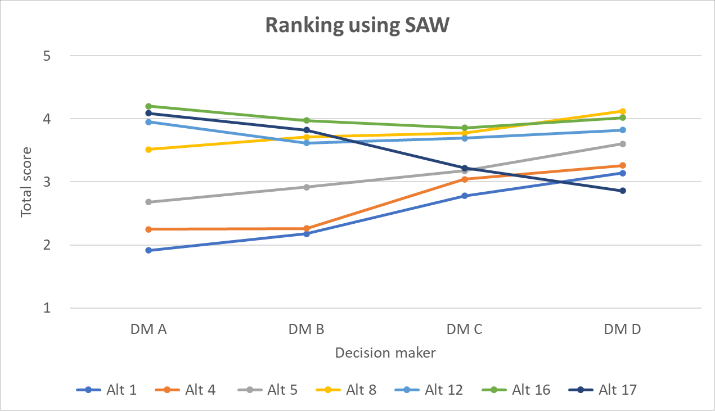 | 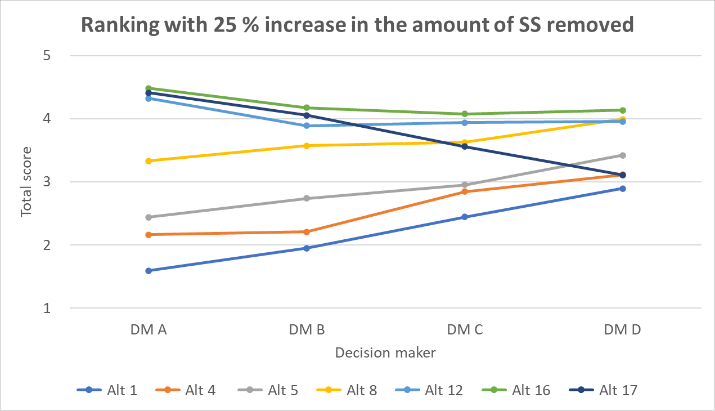 |
| (a) Ranking of alternatives with original weights using SAW (the same as Figure 5.5 (a)) | (b) Ranking of alternatives with 25% weight increase in the amount of SS removed using SAW |
|  |  |
| 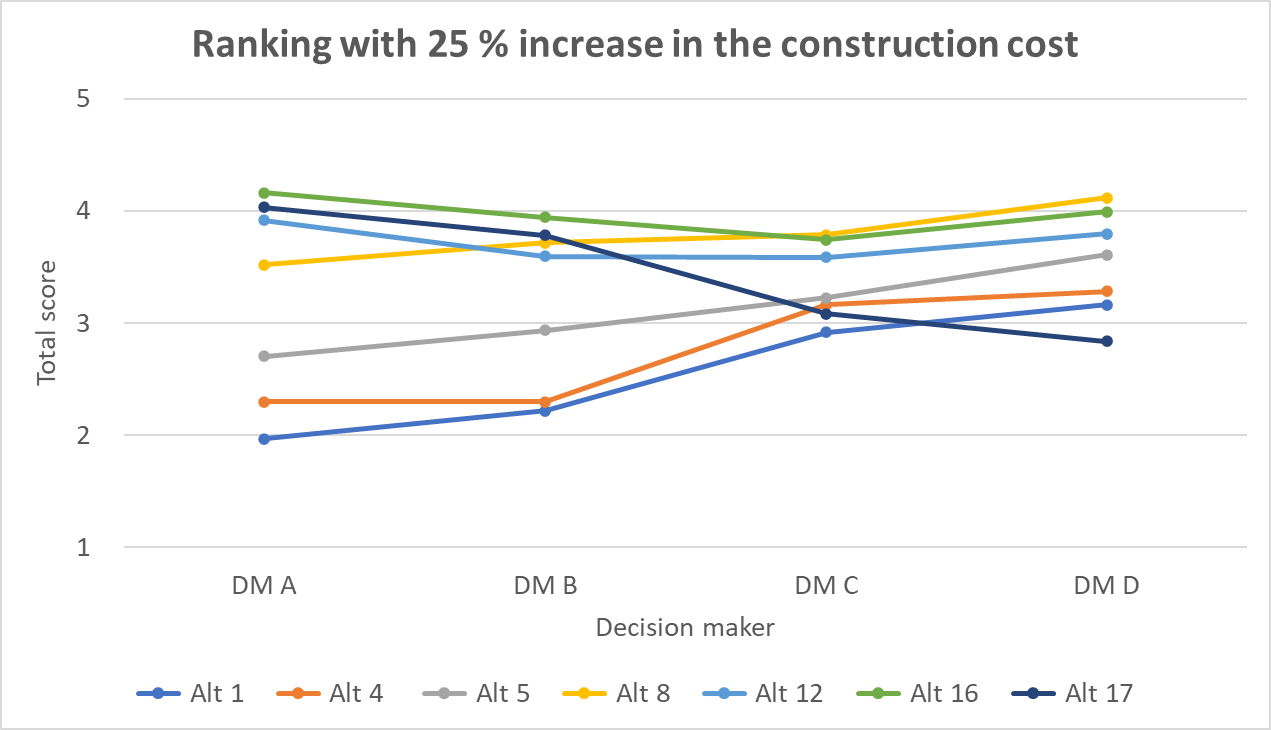 | 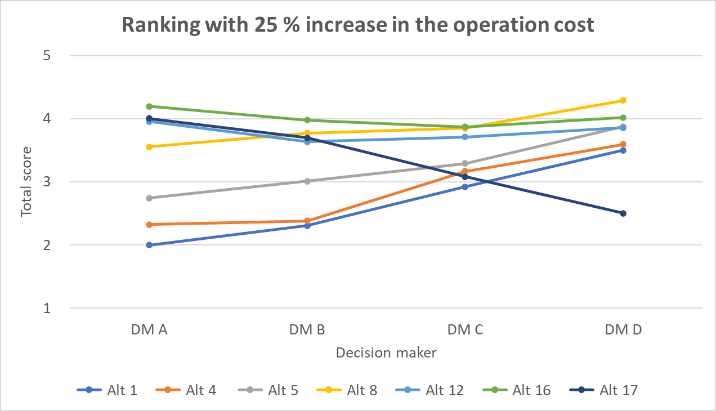 |
| (c) Ranking of alternatives with 25% weight increase in the construction cost using SAW | (d) Ranking of alternatives with 25% weight increase in the operation cost using SAW |
| 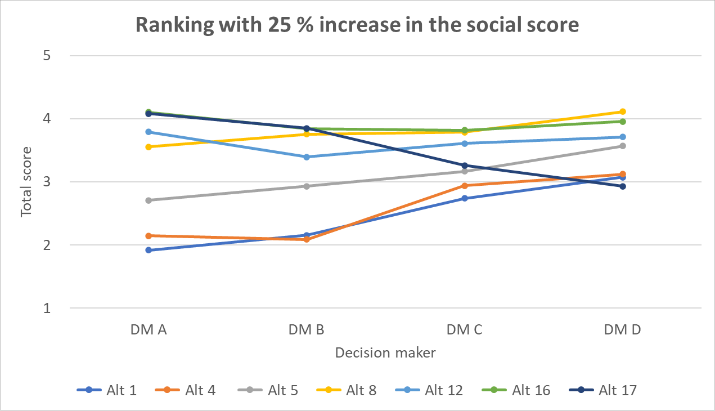 |  |
| (e) Ranking of alternatives with 25% weight increase in the social score using SAW |  |

Figure S.7. Sensitivity analysis of criteria weights using SAW

(Figure S.7 (b)), rankings of alternatives showed no change, except for alternative 8. Specifically, alternative 8 lowered from 3rd to 4th place for decision-maker B, from 2nd to 3rd place for decision-maker C, and from 1st to 2nd place for decision-maker D. Thus, in conclusion, the change in the amount of SS removed criteria did not comprehensively affect the rankings, excluding alternative 8. If the weight of the construction cost increased by 25% (Figure S.7 (c)), changes in ranking did not occur except for decision-maker C. In the case of decision-maker C, the alternatives that were previously ranked 1st and 2nd switched places; alternative 6 rose to 1st place, and alternative 16 fell to 2nd. Also, alternative 17 moved from 4th to 6th place. The rationale for this lack of significant change in decision-makers A, B, and D can be attributed to the relatively low weight (4.94-6.78%) assigned to construction cost. In contrast, decision-maker C produced a higher weight of 20.13% in construction cost, which plausibly yielded the observed changes in rankings.

If the weight of operational cost increased by 25% (Figure S.7 (d)), minimal change occurred in the rankings of alternatives, except for alternative 17. Alternative 17’s rank dropped from 2nd to 3rd for decision-maker B and from 4th to 6th for decision-maker C. Finally, if the weight of social score increased by 25% (Figure S.6 (e)), there was no reported change for decision-makers A, C, and D. For decision-maker B, however, alternative 17 emerged as the best alternative, resulting in a proximate total score to alternative 16. In addition, alternative 4 became the worst option for this party. In summary, this study demonstrated that increasing each criterion's weight by 25% did not result in significant changes in rankings. However, it was found that the change in weight in the amount of SS removed impacted the ranking of alternative 8, operational cost influenced alternative 17. To further extrapolate, the weight change in the construction cost impacted the rankings for decision-maker C, and social cost had an effect on decision-maker B.

The sensitivity analysis of each criterion is delineated in Figure S.8 in which the TOPSIS methodology for ranking was employed. If the weight of the amount of SS removed was enhanced by 25% (Figure S.8 (b)), there were negligible alterations in the rankings of the alternatives, with the exception of alternative 17. Specifically, alternative 17 exhibited a rank shift from the fourth to the third position in decision-maker C, while it increased from the seventh to the sixth position in decision-maker D. If the weight of construction cost increased by 25% (Figure S.8 (c)), there were no discernible shifts in the rankings across all decision-makers. This outcome can be attributed to the relatively low weight for construction cost, ranging from 4.94% to 6.78% for decision maker A, B, and D, so even if it is increased by 25%, the difference is not significant. The construction costs’ relatively low weight, which ranged from 4.94% to 6.78% for decision-makers A, B, and D, can explain the nominal change in rankings, even with a 25% increase in weight. In the case of decision maker B, the weight of construction cost was considerably elevated at 20.13% and the change in performance scores had a 25% increase, albeit without subsequent ranking shifts. If the weight for operational cost increased by 25% (Figure S.8 (d)), no rank shifts arose for decision makers A, B, and C. However, for decision maker D, alternative 8 experienced a rank change from the second position to the best alternative. Given a 25% increment in the weight of social score (Figure 5.8 (e)), no ranking changes occurred for any decision-makers, except for decision-maker B. In the case of decision-maker B, alternative 8 rose in rank from fourth to third, whereas alternative 4 lowered in rank from sixth to seventh.

|  |  |
| --- | --- |
| C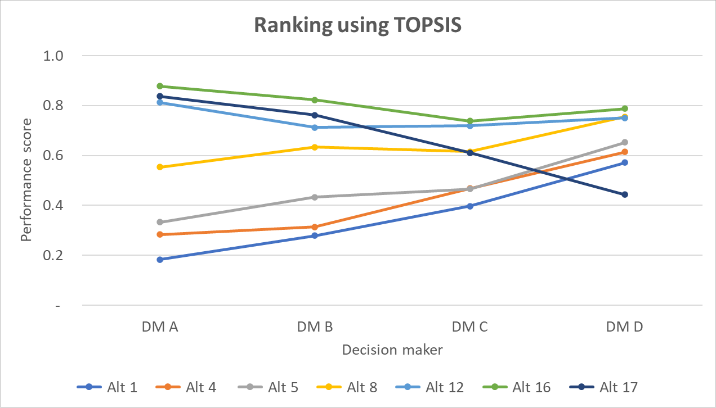 | 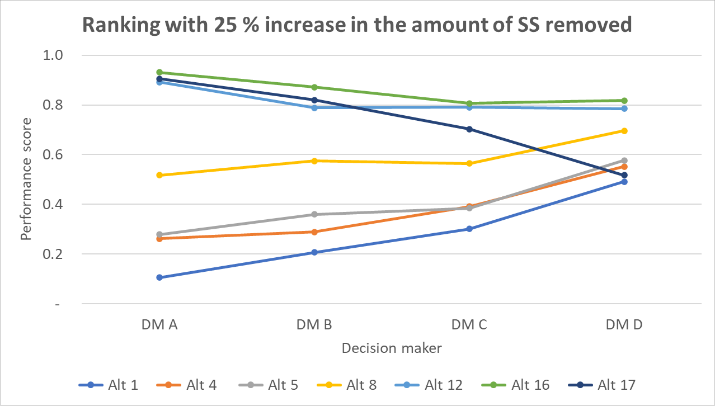 |
| (a) Ranking of alternatives with original weights using TOPSIS (the same as Figure S.5 (b)) | (b) Ranking of alternatives with 25% weight increase in the amount of SS removed using TOPSIS |
|  |  |
| 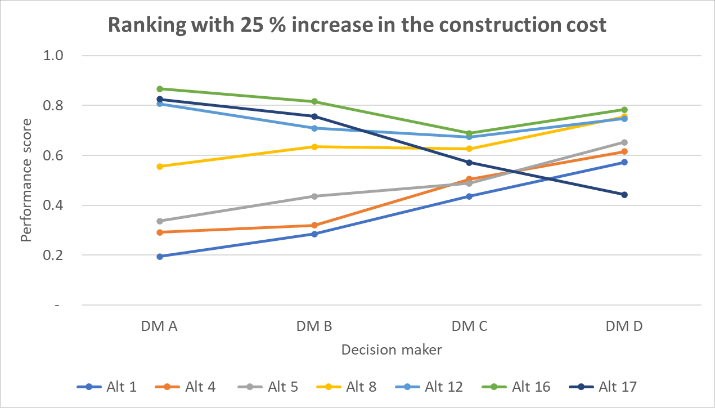 | 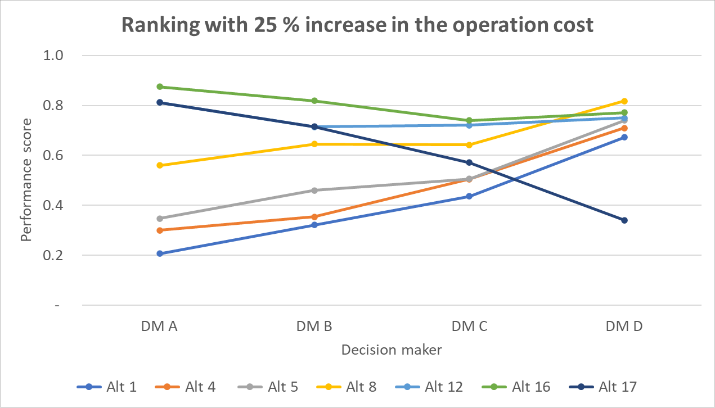 |
| (c) Ranking of alternatives with 25% weight increase in the construction cost using TOPSIS | (d) Ranking of alternatives with 25% weight increase in the operation cost using TOPSIS |
| 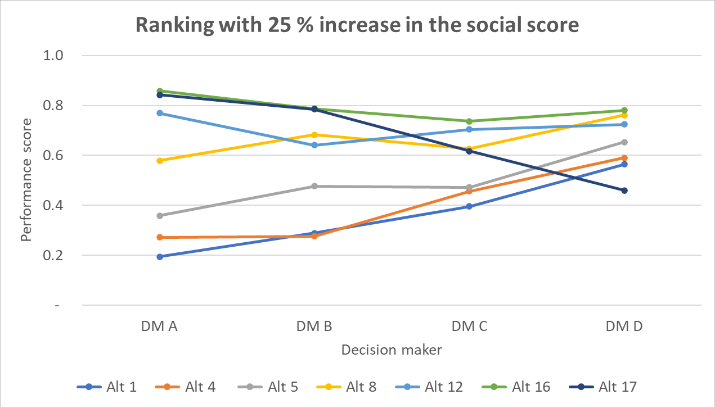 |  |
| (e) Ranking of alternatives with 25% weight increase in the social score using TOPSIS |  |

Figure S.8. Sensitivity analysis of criteria weights using TOPSIS

In summary, the sensitivity to changes in the criteria weights was relatively low when the TOPSIS methodology was employed for ranking. There were only three instances in which notable change occurred: change in the weight of the amount of SS removed influenced the ranking of alternative 17, fluctuation in the weight of the operational cost impacted the alternatives rankings in the decision maker D, and weight changes in social score were significant solely for decision maker B. In conclusion, these findings suggest that despite the inherent uncertainties regarding the decision-makers' criteria weights, the sensitivity of criteria weight in rankings was not prominent with both SAW and TOPSIS methodologies. This inference supports the idea that bar rack and grit chamber decisions can produce stable results despite the disparity of the decision makers' preferences.

#### Sensitivity Analysis Without Screening

Impact of the screening process can be examined in Figure S.9, which illustrates a comparison of alternative rankings with and without DEA application. Of the 17 alternatives assessed, seven were identified as non-inferior options and represented by solid lines, while the remaining ten are classified as inferior options and indicated by dotted lines. As depicted in Figure S.9 (b), all inferior options consistently demonstrate lower rankings. Specifically, the top three ranked alternatives unanimously fall within the non-inferior options for each decision-maker, as designated by solid lines. In conclusion, inferior alternatives are inherently negated as the best alternative under any circumstances, as at least one corresponding non-inferior alternative (reference pair) exists. In addition to this aspect, inferior options were not included in the top three ranking alternatives in this study, so it was reasonable to remove them at the screening stage.

| 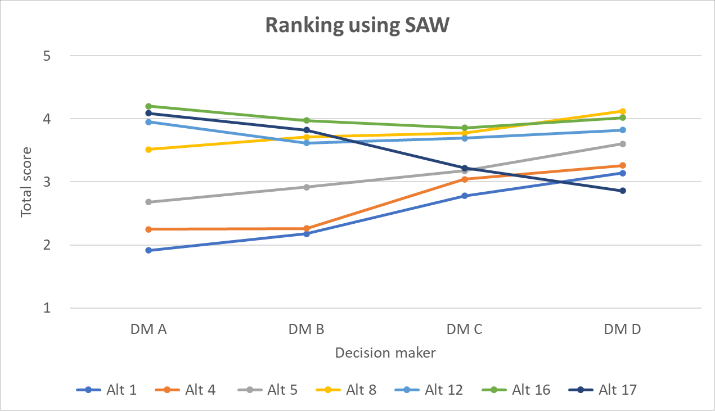 | 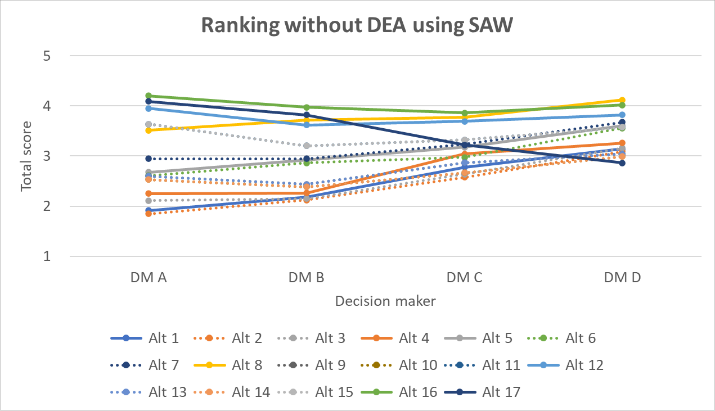 |
| --- | --- |
| (a) Ranking of alternatives with DEA screening using SAW (the same as Figure 5.5 (a)) | (b) Ranking of alternatives without DEA screening (solid lines indicate non-inferior alternatives and dotted lines indicate inferior alternatives) |

Figure S.9. Comparison of alternative rankings with and without DEA screening
